# CageCavityCalc (*C*3): A Computational Tool for Calculating and Visualizing Cavities in Molecular Cages

**DOI:** 10.1021/acs.jcim.4c00355

**Published:** 2024-07-09

**Authors:** Vicente Martí-Centelles, Tomasz K. Piskorz, Fernanda Duarte

**Affiliations:** † Instituto Interuniversitario de Investigación de Reconocimiento Molecular y Desarrollo Tecnológico (IDM), Universitat Politècnica de València, Universitat de València, Camino de Vera s/n, Valencia 46022, Spain; ‡ CIBER de Bioingeniería Biomateriales y Nanomedicina, Instituto de Salud Carlos III, Madrid 28029, Spain; § Departamento de Química, Universitat Politècnica de València, Camino de Vera s/n, Valencia 46022, Spain; ∥ Chemistry Research Laboratory, University of Oxford, Mansfield Road, Oxford OX1 3TA, U.K.

## Abstract

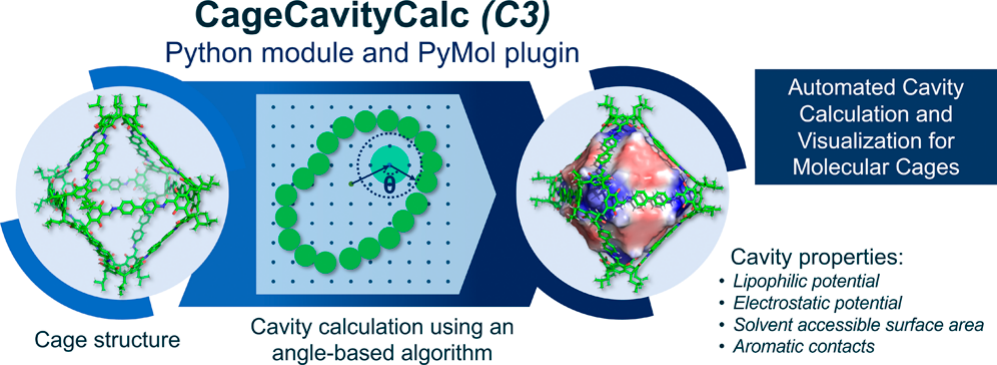

Organic(porous) and metal–organic cages are promising biomimetic platforms with diverse applications spanning recognition, sensing, and catalysis. The key to the emergence of these functions is the presence of well-defined inner cavities capable of binding a wide range of guest molecules and modulating their properties. However, despite the myriad cage architectures currently available, the rational design of structurally diverse and functional cages with specific host–guest properties remains challenging. Efficiently predicting such properties is critical for accelerating the discovery of novel functional cages. Herein, we introduce *CageCavityCalc* (*C*3), a Python-based tool for calculating the cavity size of molecular cages. The code is available on GitHub at https://github.com/VicenteMartiCentelles/CageCavityCalc. *C3* utilizes a novel algorithm that enables the rapid calculation of cavity sizes for a wide range of molecular structures and porous systems. Moreover, *C*3 facilitates easy visualization of the computed cavity size alongside hydrophobic and electrostatic potentials, providing insights into host–guest interactions within the cage. Furthermore, the calculated cavity can be visualized using widely available visualization software, such as PyMol, VMD, or ChimeraX. To enhance user accessibility, a PyMol plugin has been created, allowing nonspecialists to use this tool without requiring computer programming expertise. We anticipate that the deployment of this computational tool will significantly streamline cage cavity calculations, thereby accelerating the discovery of functional cages.

## 1 Introduction

Discrete three-dimensional (3D) organic and metal–organic (porous) cages have emerged as promising biomimetic systems.^1−4^ They offer synthetic modularity and tunability, enabling chemists to efficiently create structures with customized sizes and shapes from simple building blocks.^5−8^ A critical factor contributing to their functional properties is the presence of a well-defined inner cavity capable of binding and even catalyzing chemical reactions, prompting chemists to draw parallels between these systems and enzymes.^9−12^

In molecular cages, the affinity of the host toward a particular guest depends on various structural and electronic parameters.^13^ A key structural parameter used to assess the ability of a cage to act as a host is the relative cavity size of the cage and guest molecules. For instance, Rebek determined that “closed” organic capsules exhibit binding when the guest occupies around 55% of the host cavity volume.^14^ Since then, this rule has been extended to other supramolecular structures with varying success^15−17^ and also to enzymes.^18^

The most commonly used algorithms to calculate cavities are geometric (see description of geometric algorithms below), as they only require the Cartesian coordinates of the molecule and are therefore easy to implement.^19−21^ Based on geometric methods, several software tools have been developed for detecting, analyzing, and visualizing cavities, as analyzed in comprehensive reviews.^19−21^ These tools are primarily used to identify protein-binding pockets but have also been used to calculate the cavities of supramolecular cages.^21^ The following selected list shows examples of cavity calculation software for different types of hollow structures, such as proteins, cages, representative fragments of metal–organic frameworks, etc.: VOIDOO,^22^ McVol,^23^ Volarea,^24^ CAVER,^25^ KVFinder (including pyKVFinder, parKVFinder, and KVFinder-web),^26−29^ Fpocket (including its molecular dynamics (MDs) implementation MDpocket and FpocketWeb),^30−32^ PoreBlazer,^33^ Zeo++,^34^ CavVis,^35^ POVME,^36^ ghecom,^37^ PyWindow,^38^ MoloVol,^39^ CAST,^40^ ProteinVolume,^41^ 3 V,^42^ and Voronoia.^43^ Additionally, other software that is no longer available includes GRASP^44^ and HOLLOW.^45^ It is worth noting that VOIDOO, which is probably the most popular tool used by the supramolecular community,^46−48^ has not been updated since 1999, making it challenging to use on modern computers. For a comprehensive list of available tools, we refer the reader to comprehensive reviews that analyze cavity calculation software.^19−21^

The geometric algorithms for detecting cavities are classified into four categories based on the geometrical techniques: grid, sphere, surface, and tessellation.^19,20^ Each approach has its own strengths and weaknesses, as detailed by Simões et al.^20^ and Krone et al.^19^ Grid-based algorithms (e.g., POVME) map the atomic 3D coordinates of a molecule onto a grid and use clustering methods to identify the empty grid points that define the cavity. Sphere-based algorithms (e.g., PyWindow) use the atomic 3D coordinates and van der Waals radii of the molecule to define the molecular surface, then, the cavity is detected by scanning the surface with a probe, usually a hard sphere. Tessellation-based algorithms (*e.g*., Fpocket and CAVER) employ surface representations, such as Voronoi diagrams, to divide the molecular space into regions, each with specific cavity detection methods. Surface-based algorithms define cavities based on the solvent-excluded surface, solvent-accessible surface (SAS), or ligand-accessible surface. For detailed information on these methods, we refer interested readers to comprehensive literature reviews.^19−21^

Major challenges in detecting cavities using geometric algorithms include defining the cavity boundary, known as mouth opening ambiguity (MOA);^19−21^ grid-spacing sensitivity (GSS), which evaluates the impact of the grid voxel size; and protein-orientation sensitivity (POS), which assesses the effect of structure’s orientation within the grid.^19−21^ Simões et al.^20^ describe that grid-based methods can present GSS and POS but have no difficulties in finding cavity mouth openings. Conversely, sphere-based methods can suffer from user-assisted cavity localization (UACL), meaning the method is not fully automated and input from the user is needed, MOA, and POS. These individual techniques can be combined to mitigate or even eliminate these challenges (e.g., the KVFinder project and ghecom). For instance, GSS is solved by setting the voxel size at most half of the radius of the water probe sphere, while MOA and POS are avoided by using large probe spheres to block cavity openings. Additionally, these combined methods do not suffer from UACL as cavity detection is automated.

**Figure 1:**
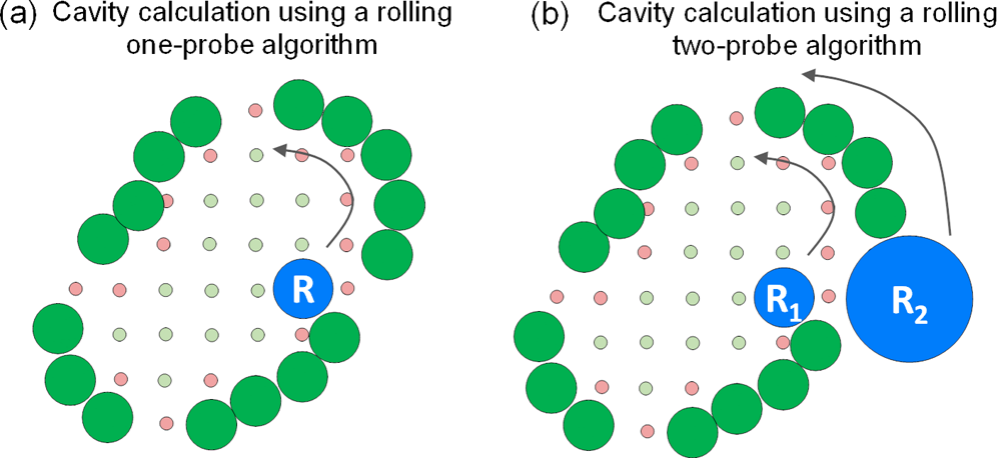
Schematic representation of geometric sphere- and grid-based methods of cavity calculation algorithms using rolling probes (R, denoted by blue spheres): (a) Rolling one-probe algorithm: The probe moves within the cavity, identifying accessible areas as cavity (green points), and if the probe collides with cage atoms, these grid points are designated as noncavity (red). The calculation fails if the probe exits the cavity. (b) Rolling two-probe algorithm: it employs an inner small probe, R_1_, and a larger outer probe, R_2_, which roll from the outside, blocking the cage windows to prevent the inner probe from exiting the cavity. However, if the radius of R_2_ is small relative to the cage windows, it may enter the cavity.

In this work, we focus on the geometric rolling probe method that relies on a combination of grid- and sphere-based algorithms. This algorithm uses one or two probes and achieves varying degrees of success in detecting cavity mouth openings (i.e., cavity windows). The one-probe algorithm is best suited for cavities with small windows, which are the apertures within a cage structure connecting its enclosed cavity with the external environment. This algorithm defines the cavity as the volume enclosed by a probe “rolling” around the entire cage structure without escaping (Figure 1a).^19−21^ On the other hand, the rolling two-probe algorithm is suitable for cavities with larger windows, solving the probing escaping from the cavity issue encountered in the one-probe method. In this approach, the cavity is defined as the volume enclosed by a small probe, which cannot escape when a second, larger probe blocks the cage’s windows (Figure 1b). However, the two-probe rolling method can fail if the radius of the larger probe is too small, allowing it to move inside the cavity through the cage windows and resulting in inaccurate volume calculations. Therefore, the user needs to carefully evaluate, and often fine-tune, the parameters. This process is laborious and difficult to generalize across different systems or when analyzing MD trajectories, where the window sizes dynamically change along the simulation time. Alternatively, one can calculate the largest sphere that fits into the cavity, as done in PyWindow, but this compromises the accuracy of the computed volume as cavities in cages often deviate from a perfect spherical shape. Therefore, efficient detection of cavity mouth openings remains a challenge for cages with large windows.

**Figure 2:**
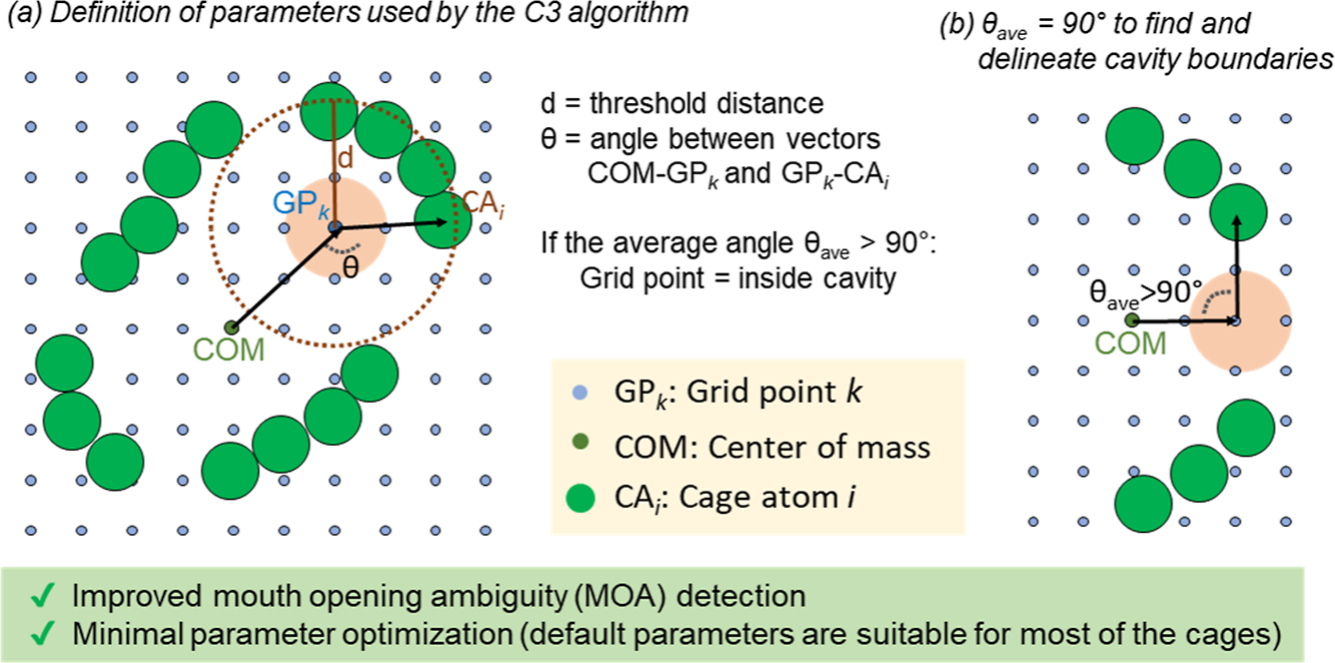
*C*3 angle-based algorithm developed herein: it uses the cavity’s angle θ between the two vectors COM-GP_*k*_ and GP_*k*_-CA_*i*_. The grid point size is automatically determined by the grid resolution.

To address the challenge of detecting cavity mouth openings (i.e., cavity windows), taking the concept of the rolling probe algorithm, we developed a sphere- and grid-based method that uses a geometric algorithm based on a novel angle measurement technique to determine the cavity boundaries (Figure 2). This method relies on determining the angle θ formed by the center of mass (COM) of the cage, the probe, and the atom of the cage. For probes that do not overlap with cage atoms, the algorithm calculates the average of all angles θ for each cage atom within a certain threshold distance, which is automatically calculated as the diameter of the maximum escape sphere from the cavity. If the average angle is greater than 90°, the probe is considered to be inside the cage; otherwise, it is considered to be outside of the cavity. This algorithm is specifically designed to calculate the internal cavity volume of molecular cages. The cavity is calculated in an automated manner and therefore not affected by UACL. In this work, we test the efficiency of the developed *C*3 angle-based algorithm and its performance on MOA, GSS, and POS.

In addition to host–guest size complementarity, guest binding is significantly influenced by electrostatic,^49,50^ hydrogen bonding,^51^ and van der Waals interactions^49,52^ with the host, which can be significantly affected by the solvent.^53^ For example, in water, the hydrophobic effect plays a key role in driving guest binding.^54−56^ Ward and co-workers demonstrated that hydrophobic guests exhibit 2–3 orders of magnitude higher association constants compared to polar guests.^57,58^ Similarly, Ballester and co-workers established a linear relationship between binding free energies and the surface area of the nonpolar guests’ fragments, indicating a hydrophobic effect of 33–38 cal mol^–1^ Å^–2^.^59^

Electrostatic complementarity is often visualized through electrostatic potential (ESP) surfaces.^60,61^ Molecular ESP can be calculated using ab initio methods or from charge distributions obtained by numerically solving the Poisson–Boltzmann equation or its approximate form, the generalized Born model.^62,63^ Additionally, hydrophobic interactions can be indirectly estimated using methods based on hydrophobic–lipophilic interactions, which quantify the tendency of nonpolar molecules to avoid contact with polar solvents, inducing aggregation and self-coiling.^64,65^ Another parameter, molecular hydrophobicity potential (MHP), derived from the 1-octanol/water partition coefficient (logP),^66,67^ has proven useful in describing hydrophobicity in proteins,^68^ analyzing protein pockets in docking engines, and predicting protein–ligand binding affinities.^69−72^ However, despite its utility in ligand–protein binding analysis, the MHP descriptor has not been commonly used to analyze cage cavities, likely due to the lack of user-friendly tools for their generation.

In this work, we introduce *C*3, a tool that calculates the cavity-size electrostatic and hydrophobic potentials of molecular cages. The developed Python module can be used from the command line, in a python script, or as a plugin to the molecular visualization program PyMol.^73^ Overall, the developed module enables the efficient characterization of cavity and host–guest properties. Herein, we outline the methodology and demonstrate the ability of *C*3 to determine the cavity volume of molecular cages with diverse sizes and shapes. We illustrate the utility of *C*3 and highlight its advantages in terms of accuracy compared to state-of-the-art methods when assessing 16 cavities with different topological and morphological features. Furthermore, we employ *C*3 to compute the MHP and ESP surfaces in the calculated cavities, aiding in the determination of host–guest properties.

**Figure 3:**
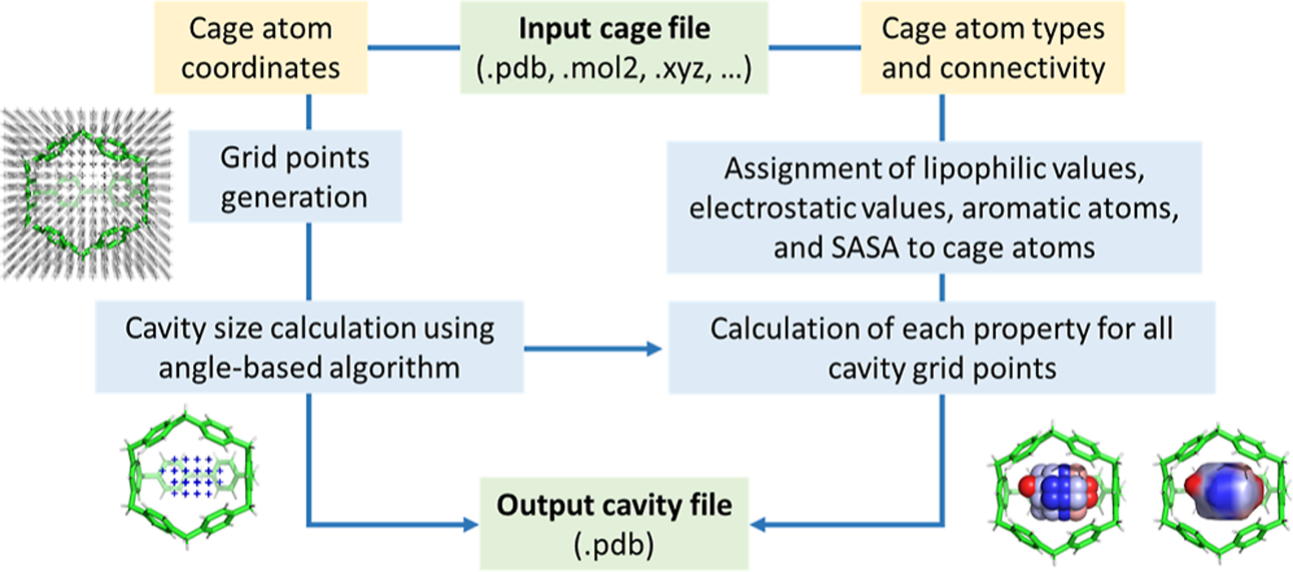
*C*3 workflow to determine the cage cavity and the properties of each cavity grid point.

## 2 Results and Discussion

### 2.1 Software Outline

*C3* is a stand-alone Python module designed for the characterization of organic and metallocage cavities. *C*3 can be installed using the pip package manager by running the command “pip install CageCavityCalc”. The calculations in this article have been performed using *C*3 version 1.0.5. Detailed installation instructions are provided in Supporting Information §3. *C*3 is compatible with Windows, macOS, and Linux, requiring Python 3.7 or later. It employs scientific programming libraries, including NumPy (v1.26.4),^74^ SciPy (v1.13.0),^75^ and scikit-learn (v1.4.2).^76^ Optional functionalities can be enabled using the chemical programming libraries RDKit (2023.09.5),^77^ MDAnalysis (v2.7.0),^78^ OpenBabel (v3.1.0),^79^ and Cgbind (v1.0.3),^80^ which facilitate the handling of chemical structures, including the identification of functional groups and the calculation of molecular properties, or the generation of cage structures from ligands and metals (i.e., using *Cgbind*, as shown in Supporting Information section S1).

The core of the code is based on the *Cavity* class, which features several functions: file reading, COM calculation, 3D grid setup, identification of grid points within the cage, calculation of MHP and ESP, and the subsequent saving of results in PDB and/or PyMol formats. Additionally, the *GridPoint* and *CageGrid* classes define attributes associated with each grid point in the 3D grid. The code also includes different functions for assigning hydrophobic values to the cage atoms, computing partial charges of the cage atoms, and calculating the maximum radius escape sphere, among other functions (see Supporting Information §S1 for a full description). Additionally, it offers a PyMol plugin (PyMol 3.0.0)^73^ with the corresponding *C*3 graphical user interface (GUI) to set up all calculation parameters of *C*3 (see details in Supporting Information §S2). Figure 3 illustrates a simplified schematic diagram of *C3* functionality.

Upon loading the 3D cage structure into *C*3, a 3D grid filled with grid points is generated, with customizable size and grid spacing (default parameters are described in Supporting Information §S2). The cavity size is then calculated by iteratively examining all grid points to determine whether they form part of the cage cavity.

Once the cavity size is calculated, several properties are computed using the molecular properties of each cage atom. These properties include aromatic contacts, SAS area (SASA), hydrophobicity (MHP), and/or ESP. The calculated values can be saved as B-factor in the generated cavity PDB file, facilitating their visualization in any standard molecular visualization software; one property is saved per PDB file. Alternatively, users can calculate multiple properties using the PyMol plugin, which enables storing all computed properties in the same PyMol session file. This feature enables users to interactively select the properties they want to visualize and save a PDB file for each of them. The following sections detail each of these steps.

### 2.2 Overview of the Software

#### 2.2.1 Loading of the Cage Structure

The first step in *C*3 involves loading the Cartesian coordinates of the cage structure obtained by the user from either a crystal structure (CIF files) or via molecular modeling, employing software such as Stk^81^ or Cgbind.^82^*C3* supports various molecular file formats such as .xyz, .pdb, .mol, .mol2, and others handled by MDAnalysis.^78^ The plugin stores the atom types and the Cartesian coordinates in NumPy arrays.

#### 2.2.2 Cavity Volume Calculation

In *C*3, the cavity volume calculation is performed without requiring user-defined parameters as the default settings yield satisfactory results across a wide range of cages. The algorithm starts by generating a box around the cage structure with dimensions to fit all cage atoms, followed by the generation of a 3D grid. By default, the grid spacing is set to 1 Å, allowing fast running times for small- and medium-sized cages (in this work, we categorize cages with approximately 300–400 non-H atoms, or 25–30 Å cage diameter as medium-sized). Users can adjust grid spacing based on computational resources. Typically, a finer grid (smaller spacing, typically 0.5 Å) is used for smaller cages, while a coarser grid (larger spacing, typically from 2 to 4 Å) is used for larger cages. The calculation of the cavity volume involves iterating over grid points and classifying them as either part of the cavity or outside of it. This selection involves two steps:

(a) Locate all cage atoms within a threshold distance from the selected grid point. By default, the threshold is set to two times the window size, which is obtained from the diameter of the maximum escape sphere from the cavity,^82^ ensuring that cages with large windows are correctly processed without manual adjustments.
(b) For each grid point, the cavity angle θ is calculated as the angle between two vectors: the vector defined by the COM of the cage and the selected grid point *k* (COM-GP_*k*_), and the vector defined by the selected grid point *k* and the atom of the cage *i* (GP_*k*_-CA_*i*_) (see description of the angle in Figure 2a). The θ angle is calculated for each cage atom *i* in the threshold distance. Then, all θ cavity angles are averaged to obtain θ_average_; if the average cavity angle θ_average_ is larger than 90°, the atom is considered inside the cage; otherwise, it is considered outside the cavity.

The process of iterating over all cage atoms within the threshold distance is sped up by using the SciPy Spatial KDTree algorithm, which partitions multidimensional data into a binary tree structure, enabling efficient nearest-neighbor searches and spatial queries.^75^ To achieve this, the Cartesian positions of all cage atoms are stored in a KDTree dictionary by atom types, significantly reducing the time required for obtaining atoms within a threshold distance compared to brute-force iteration. Once the iteration over all grid points in the grid is completed and all grid points are assigned as within or outside the cavity, *C*3 uses a DBSCAN clustering algorithm to identify the main cavity region, disregarding isolated grid points that do not correspond to the cavity.^83^ In cases where more than one cavity is identified, the main cavity is selected based on the largest cluster, or alternatively, the cluster closest to the cage COM.

**Figure 4:**
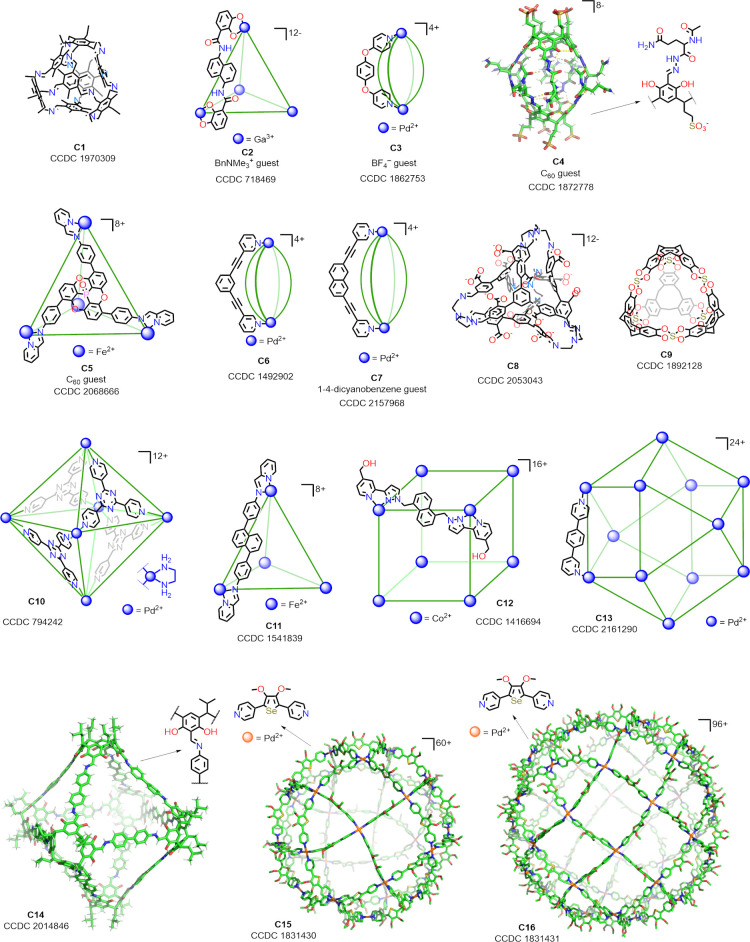
Data set used to test the efficiency of *C*3. The structures for **C1**–**C16** cages were extracted from the corresponding CIF files obtained from the CCDC.

To demonstrate the utility of the *C3* algorithm, we created a data set of 16 cages (**C1–C16**, Figure 4) extracted from the Cambridge Structural Database.^84^ The data set includes cages with different topological and morphological features, including cages with well-defined closed cavities or medium-sized windows (**C1–C12**), cages with inclusion complexes (**C2–C5**), and cages with large windows (**C13–C16**).

When computing cavity volumes, the grid spacing and distance threshold for the 90-degree calculation must be carefully chosen to balance computational cost and accuracy. The grid spacing was set to 0.5 Å for cages **C1–C12** and 1.0–3.5 Å for cages **C13–C16**. As expected for a geometric algorithm, *C*3 is sensitive to grid spacing. For example, increasing grid spacing from 0.5 to 1 Å in cage **C1** led to smaller computed volumes because the coarser grid (i.e., larger grid spacing) cannot capture the finer cavity details. To evaluate the grid-spacing effect on the computed volume, we computed the cavity volume of cages **C1**–**C16** using different grid spacings (see Figure S2 in Supporting Information §S6), observing that cavity volume is more sensitive to grid spacing for smaller cavities (e.g., **C1** and **C5**). We observed that reducing the grid spacing results in a convergence of the computed cavity volume that depends on the cavity size, with larger cavities achieving convergence more easily (see Figure S2 in Supporting Information §S6).

We tested MOA using cages with small, medium, and large windows. We observed that *C*3 performs well in automatically identifying and delineating cavity boundaries (see Supporting Information §S7). The default distance threshold for the 90-degree calculation, set at 2.0 times the window size, works well for cages with completely closed cavities or medium-sized windows (**C1–5**, **C8**, and **C11**) and cages with significant openings (**C6**, **C7**, **C9**, **C10**, and **C12**). However, for cages with larger openings (**C13–16**), it is necessary to increase the threshold to 3.0–4.5 to obtain satisfactory results and prevent the probe from “escaping” the cavity. In such cases, users need to visually inspect the computed cavity and adjust the distance threshold for the 90-degree calculation as needed. If the probe is escaping the cavity through the window, increasing the distance threshold is recommended. Conversely, if the calculated cavity looks spherical with an underestimated volume, the distance threshold needs to be reduced.

Finally, we tested the POS of *C*3 by randomly rotating the XYZ coordinates of the cages. For this, we generated five structures with random rotations for each cage **C1–C16**. We calculated the volume of each rotated cage structure using the same parameters and determined the mean volume and standard error (Table S3 in Supporting Information §S5). The computed mean volumes have a relative error of 0.9% relative to the mean volume, ranging from 0.2% for **C4** and **C11** to 2.8% for **C10**. Overall, comparing *C3*‘s performance on POS (Table S3), GSS (Figure S2), and MOA (Figure S3), we observed that cavity volume is more affected by grid spacing (GSS) than MOA and POS. This highlights the importance of carefully setting up grid spacing by the user.

**Figure 5:**
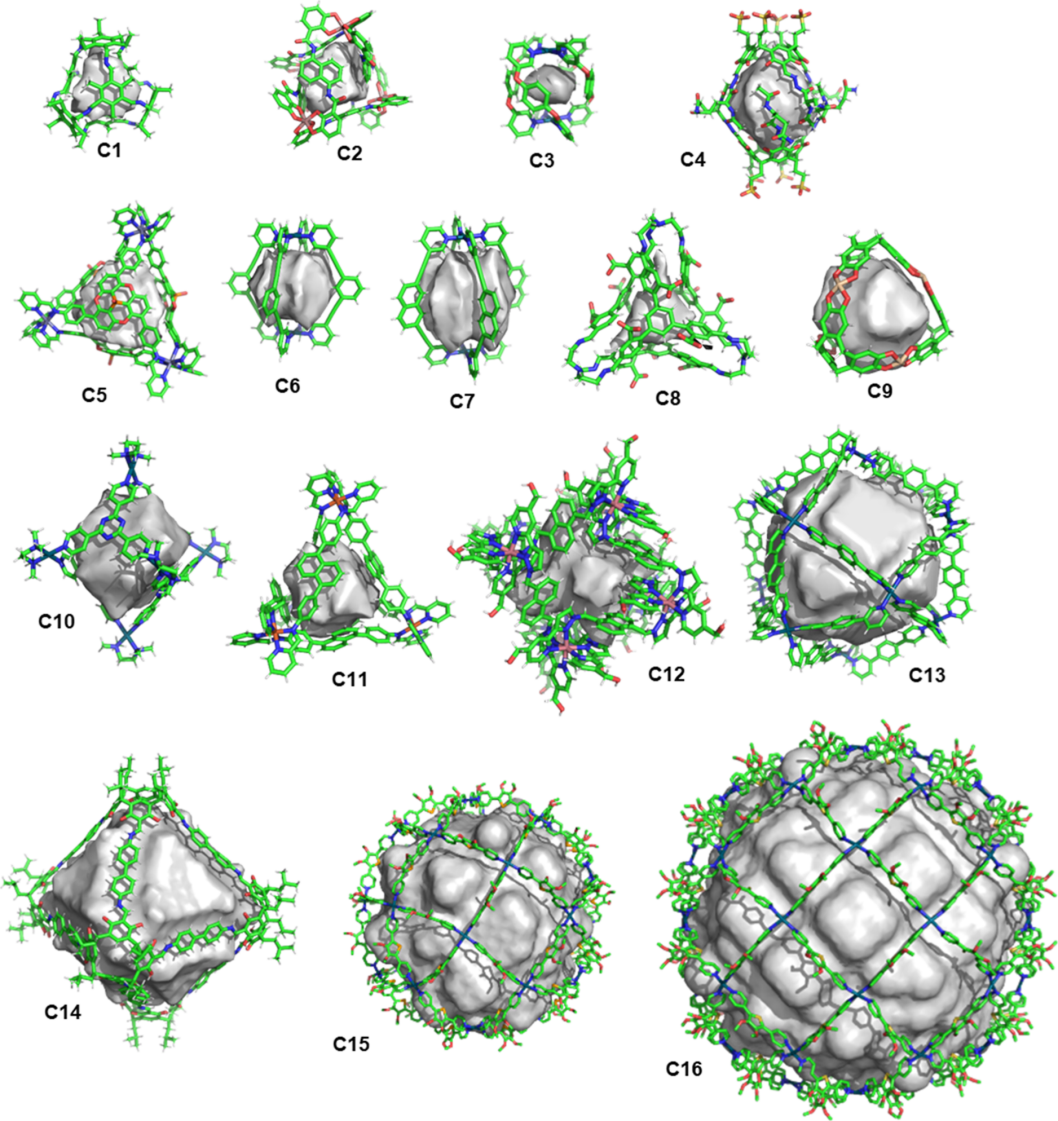
Computed cavities with *C*3 for molecular cages **C1**–**C16**.

The cavity volume results obtained using *C*3 for cages **C1–16** are depicted in Figure 5 and Table 1, with the parameters described in Table S2 in Supporting Information §S4. Initially, we computed cavity volumes of cages **C1–C16** using the default parameters of grid spacing 1 Å (except for cages **C15** and **C16**, which, due to hardware limitations, required larger grid spacing of 3 and 3.5 Å, respectively) and a distance threshold for the 90-degree angle of twice the window size. We then refined the computed volumes by optimizing the parameters. This included setting grid spacing to 0.5 Å for cages **C1**–**C12** and adjusting the distance threshold for the 90-degree calculation (in window size units) to 3 for cages **C13** and **C14** and 4.5 for cages **C15** and **C16**. Comparing the cavity volumes computed with the optimized and default parameters shows a mean relative absolute error (MRAE) of 26.7%, indicating that the default parameters provide reliable cavity volumes.

We also compared these results to those obtained using eight different cavity characterization software (KVFinder, Fpocket, MoloVol, CAVER, ghecom, pywindow, and POVME) recently studied by Lopes-de-Oliveira, György Szalóki, and co-workers,^21^ and VOIDOO, widely used by the supramolecular community. The results are summarized in Table 1, and further details regarding cavity calculation parameters can be found in Supporting Information §S7. As this data set contains cages without guests and open-cavity cages with guests, for these cages, it is not possible to obtain the cavity volume estimates using Rebek’s 55% rule. Therefore, to assess the relative performance between software, we obtained the cavity median volume for each **C1–C16** cage from the computed volumes with all tested software. The median was chosen instead of the mean as a better representation of the central tendency due to the non-normal distribution and the presence of outliers in the data. Subsequently, we calculated the MRAE relative to the median for each software and cage, i.e., MRAE_Cage_ = the absolute value of 100 × (volume – median volume)/median volume. MRAE essentially measures the discrepancy between a software’s calculated cavity volume and the median volume, expressed as a percentage. The obtained MRAE values were in accordance with literature data,^21^ showing large outliers (MRAE_Cage_ > 80%) for most of the cases for the computed volumes with the VOIDOO software, while *C3* yielded the lowest MRAE (16%), with a maximum MRAE_Cage_ of 45% obtained for cage **C1**. Remarkably, it highlights the the accuracy of its cavity calculation algorithm.

To validate the accuracy of *C3*’s cavity detection algorithm, we compared the performance of *C3* against cavity volumes obtained from Rebek’s 55% rule (i.e., cavity volume = guest volume/0.55) for the inclusion complexes of Benchmark Data set 1 reported by Guerra et al.^21^ This data set was specifically designed with cages with well-defined and closed cavities to evaluate the performance of cavity calculation software. The cavity size values obtained from Rebek’s rule show a good correlation with the *C*3 computed cavity volume, with an MRAE of 22.6% (see Table S11 in Supporting Information §S9). This shows that *C*3 can properly assess volume estimation with performance close to that obtained with the KVFinder project (MRAE = 16.1%) and Fpocket (MRAE = 16.9%).^21^ It is important to note that this rule is valid for cages with a rigid and closed cavity, and therefore deviations are expected with flexible open cages that may still allow a guest to bind even if cavity occupancy by the guest deviates significantly form the 55% rule.^3,13^

**Figure 6:**
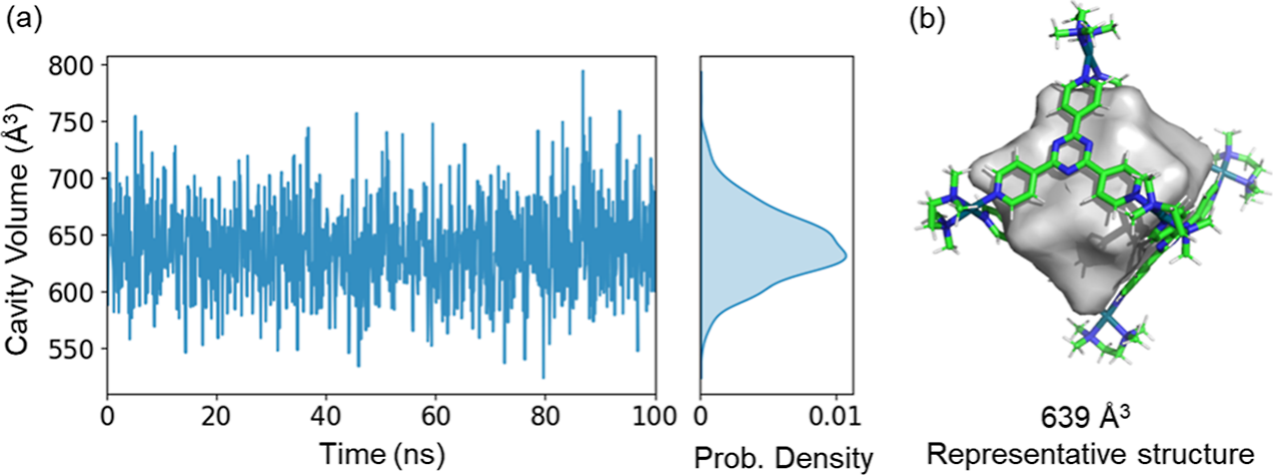
Analysis of the volume change during a 100 ns MDs trajectory of cage **C10** using *C*3 (cavity calculation parameters can be found in Supporting Information §S4). (a) Evolution of the volume over time and the corresponding probability distribution of cavity volumes. (b) Representative structure having a volume close to the mean cavity volume.

An important feature of our algorithm is its compatibility with MD simulations, enabling the assessment of dynamic changes in volume over time. To illustrate this, we analyzed the MD trajectory of cage **C10**, which provides a robust and well-defined cavity with a volume variation over time of *ca*. 10% (*V*_cavity_ = 638 ± 38 Å^3^, see Figure 6). These results highlight the robustness of the angle-base algorithm of *C*3 for analyzing MD trajectories.

We showed that the *C*3 algorithm is efficient in calculating cavity volumes on cages with all types of shapes, cavity sizes, and window sizes. The angle-based algorithm of *C*3 is especially efficient with cages with large windows, where the rolling probe algorithm fails due to the probe escaping from the cavity. Once the cavity calculation is completed, various properties of the cavity are computed for a more comprehensive understanding of the cavity’s characteristics and the interaction with potential guest molecules, thereby aiding in enhancing and designing novel host–guest complexes. These properties include aromatic contacts, SASA, hydrophobicity (MHP), and/or ESP, as described below.

#### 2.2.3 Hydrophobic Potential of the Cavity

The hydrophobicity of the cage cavity serves to identify favorable interactions with nonpolar guests in aqueous media. This is achieved by assigning hydrophobic contributions (*F*_*i*_) to each atom in the cage, which were tabulated by Ghose and co-workers for various atom types based on their contributions to the 1-octanol/water partition coefficient.^100^

Subsequently, the MHP is computed for each grid point within the cavity. MHP represents the cumulative hydrophobic contributions (*F*_*i*_) from all neighboring *N* atoms, weighted by a distance function *f*(*d*_*ik*_)
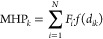
1

The commonly used distance functions are:
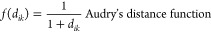
2
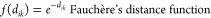
3
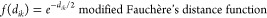
4

These functions exhibit maxima at zero, followed by decay as distance increases, reflecting the diminishing hydrophobic influence from distant atoms. Audrey’s function attempts to simulate the decay mimicking Coulomb interaction (eq 2). Despite lacking a physical foundation, it has proven useful for generating informative visualizations. Fauchère proposed an alternative exponential decay function based on observed proximity effects during octanol/water partition calculations (eq 3). Both Audry’s and Fauchère’s distance functions do not appear to be adequate beyond the SAS, and the slightly modified Fauchère’s distance function (eq 4) overcomes this limitation.^101−103^

The hydrophobic index (HI) is defined based on the distinction between negative MHP values (MHP^–^) associated with polar regions and positive MHP values (MHP^+^) corresponding to hydrophobic regions, as shown in eq 5.^69,70^
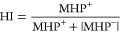
5

**Figure 7:**
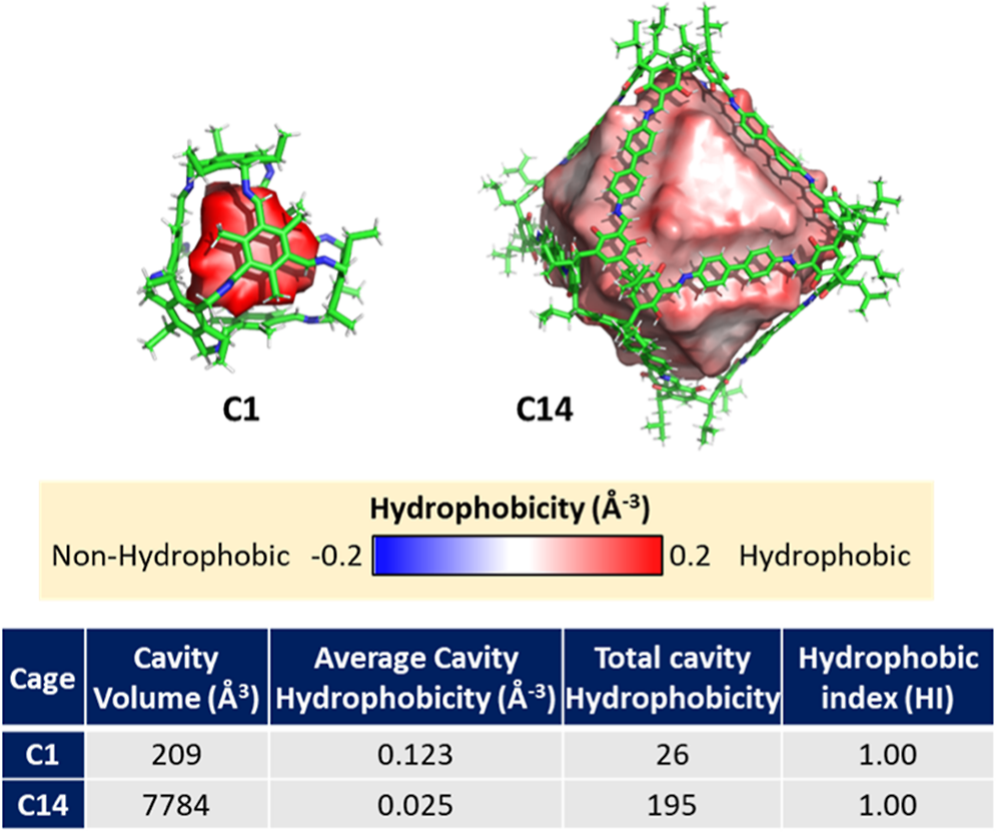
Calculation of the hydrophobicity of cages **C1** and **C14** using the Ghose method and Fauchere distance function. Cavity calculation parameters are described in Table S2.

The calculated hydrophobic potential information is then stored as B-factor data for each cavity grid point alongside the original cage coordinates in a PDB format. This format enables users to visualize these values easily using standard visualization software such as Chimera, VMD, or PyMol (Figure 7).

To illustrate the use of *C*3 for calculating cavity properties, we computed the MHP for cages **C1** and **C14**. The average cavity hydrophobicity, 0.025 for **C14** and 0.123, for **C1**, indicates that the cavity of **C1** is *ca*. 4.9 times more hydrophobic than **C14** (Figure 7). This illustrates that small cavities efficiently isolated by the cage walls are much more hydrophobic than large cages containing large windows. However, the algorithm is unsuitable for calculating the hydrophobicity of metallocages due to the lack of tabulated hydrophobic contributions for metals.

#### 2.2.4 ESP of the Cavity

The ESP is a key parameter to understand the nature of the interactions within a molecular cage and predict the interaction with potential guests, particularly polar guests. We have implemented the calculation of the ESP in *C*3 by mapping the partial charge contributions from the cage atoms onto a grid representing the cavity (eq 6)
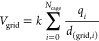
6where *k* is the Coulomb constant, *q*_*i*_ is a partial charge of a cage atom, and *d*_(grid,*i*)_ is the distance between the cage atom and the cavity grid point. The partial charges are determined using the electronegativity equalization method (EEM) implemented in Open Babel,^79^ a widely accepted and efficient procedure for deriving charge-based descriptors in QSAR studies.^104−106^

**Figure 8:**
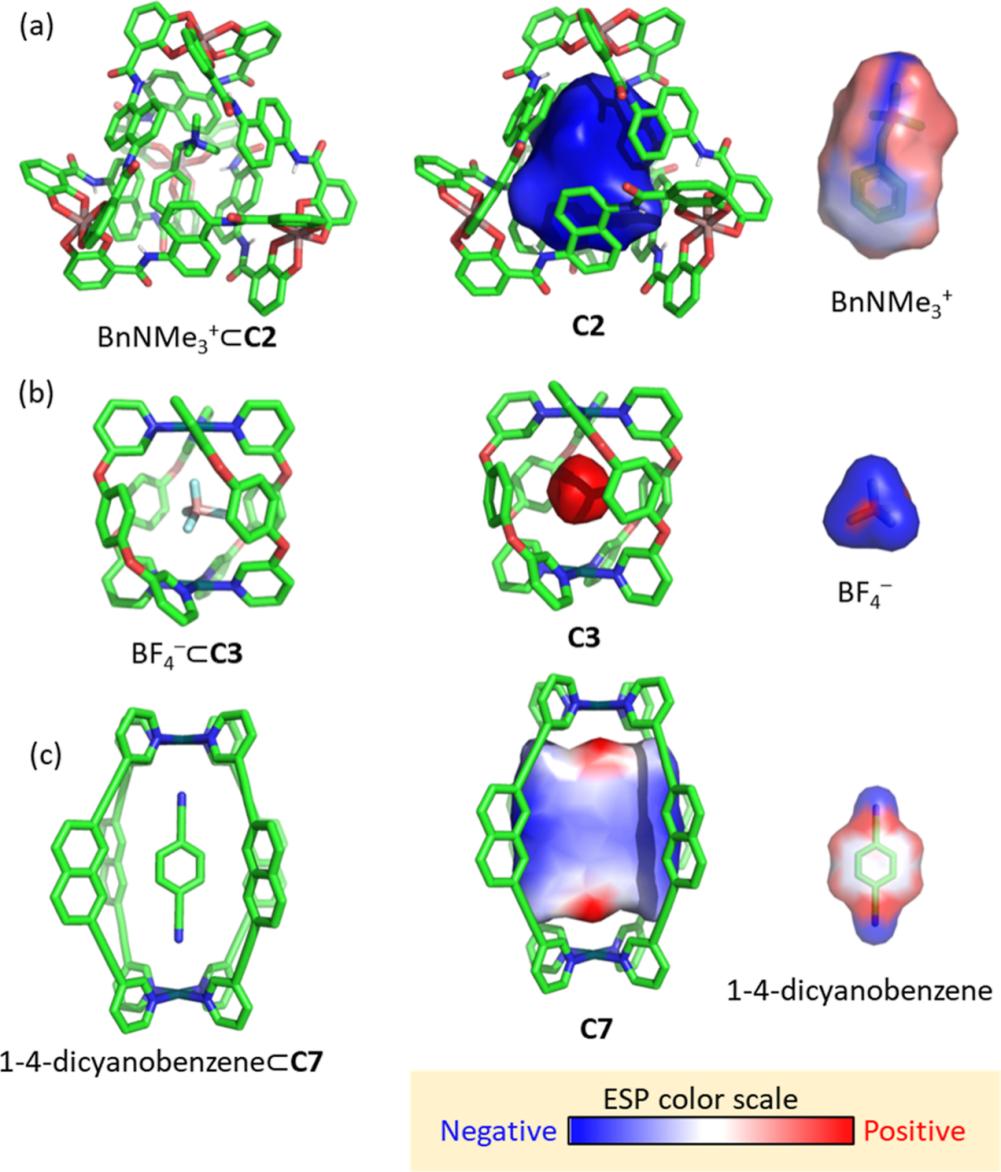
Crystal structure of host–guest complexes, ESP mapped on the cavity of empty cages, and partial charge mapped on the van der Waals sphere of guests were calculated using the EEM implemented in OpenBabel. (a) Cage **C8** with the BnNMe_3_^+^ guest. (b) Cage **C3** with the BF_4_^–^ guest. (c) Cage **C7** with the 1,4-dicyanobenzene guest.

The cavity ESP is a valuable tool for identifying favorable interactions between hosts and polar guests. Cationic and anionic cages often encapsulate their respective counterions, as illustrated by complementary electric potentials of cation and ions for the **C2** cage and BnNMe_3_^+^ complex (Figure 8a) and the **C3** cage and BF_4_^–^ complex (Figure 8b). Such analysis is also useful for neutral guests, as exemplified by the computed ESP for cage **C7** and 1,4-dicyanobenzene as potential guests. The complementarity of their ESPs suggests the formation of a strongly bound host–guest complex (Figure 8c).^91^

### 2.3 PyMol Plugin

**Figure 9:**
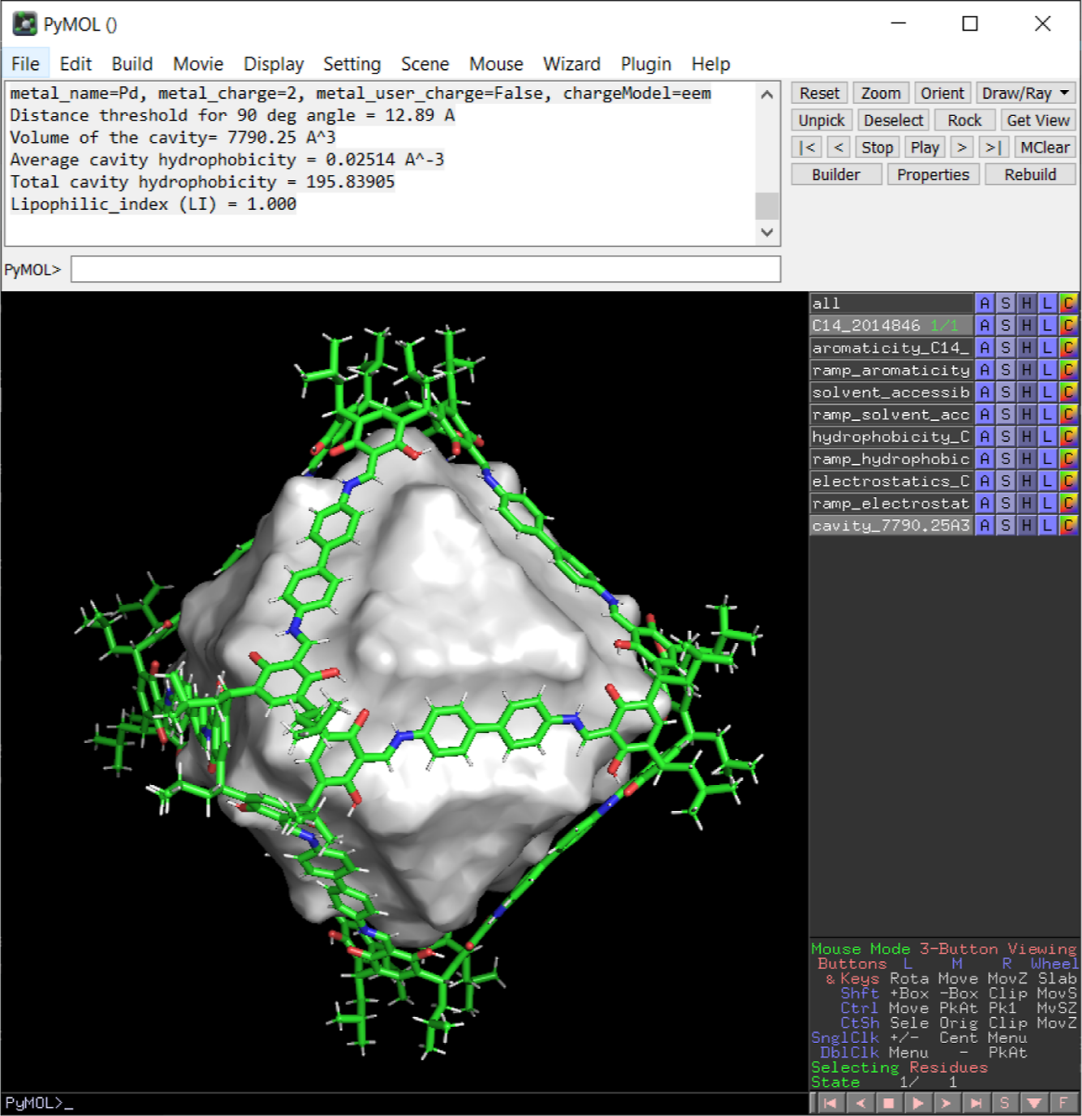
Screenshot of the results after running the *C3* PyMol plugin.

In addition to the Python module and the command-line interface, we have developed a GUI as a PyMol plugin that requires no programming skills. This GUI enables users to select a molecule and perform calculations with just a few clicks. To use it, the user simply needs to load the molecule in PyMol, open the *C*3 plugin, select the molecule from the drop-down list, and then press the “Calculate volume” button. Optionally, the user can adjust the grid size, select the properties to calculate (and adjust their parameters, such as the hydrophobicity method, the distance function, etc.), or choose a clustering algorithm to remove noisy cavity points, i.e., to remove isolated cavity points that do not belong to the main cavity. As an example, we provide the output obtained for the calculation of cage **C14**, providing in the PyMol session the output obtained for the cavity, with an individual output for each selected property (Figure 9).

**Table 1:** Calculated Cavity Volumes with *C3* and State of the Art Cavity Calculation Software (Å^3^)

cage	CCDC	median	*C*3	KVFinder project	Fpocket	MoloVol	CAVER	Ghecom	pywindow	POVME	VOIDOO	refs
**C1**	1970309	144	209	217	144	186	223	127	89	69	64	(85)
**C2**	718469	269	295	269^a^	279^a^	289^a^	339^a^	192^a^	90^a^	108^a^	101	(86)
**C3**	1862753	44	63	78^a^	84^a^	44^a^	65^a^	29^a^	37^a^	11^a^	14	(87)
**C4**	1872778	704	726	731^a^	805^a^	747^a^	682^a^	704^a^	517^a^	409^a^	396	(88)
**C5**	2068666	627	699	737^a^	627^a^	650^a^	742^a^	606^a^	532^a^	319^a^	447	(89)
**C6**	1492902	254	308	114	b	434	392	305	134	202	15	(90)
**C7**	2157968	403	496	384	b	619	448	422	174	355	26	(91)
**C8**	2053043	100	130	104	75	105	135	100	77	31	35	(92)
**C9**	1892128	617	672	558^a^	617^a^	648^a^	659^a^	669^a^	412^a^	373^a^	38	(93)
**C10**	794242	508	667	497	652	638	508	656	293	412	95	(94)
**C11**	1541839	461	458	488^a^	495^a^	526^a^	543^a^	461^a^	314^a^	211^a^	91	(95)
**C12**	1416694	613	613	866^a^	542^a^	984^a^	1008^a^	936^a^	263^a^	275^a^	117	(96)
**C13**	2161290	5240	5240	6849	4190	8641	8334	7933	4252	5021	867	(97)
**C14**	2014846	8073	7784	8336	8073	8997	8420	9051	5740	7973	1478	(98)
**C15**	1831430	37,728	31,327	37,728^a^	39,077^a^	62,843^a^	22,941^a^	59,147^a^	30,568^a^	45,704^a^	14,310	(99)
**C16**	1831431	62,568	65,722	52,474	47,480	121,832	10,687	49,342	62,568	86,464	92,290	(99)
MRAE (%)			16	20	27	32	33	21	33	38	70	

a Values from ref (21).

b No internal cavity was obtained.

## 3 Conclusions

We have introduced an automated cavity calculation software, *C*3, deployed as a Python module, for calculating cage cavities, as well as aromatic contacts, SASA, hydrophobicity (MHP), and/or ESP. The angle-based method that we developed offers a practical, easy-to-implement, and computationally efficient method, eliminating the need for parameter adjustments in most cases. However, results can be improved by fine-tuning the distance threshold for the 90-degree grid spacing. This approach facilitates automated analyses across cages of different sizes, shapes, and window dimensions. Users can vary the grid spacing to achieve the desired cavity resolution and optimize calculation time. Typically, smaller grid spacing is suitable for small cages, while larger spacing is preferable for large cages.

An important feature of our method is the improved performance in cages with large windows, providing improved MOA detection. The method was benchmarked on a wide range of cage structures with different topological and morphological features, including cages with well-defined closed cavities, inclusion complexes, and cages with large windows. The main advantages of our method are its easy use and general applicability to a wide range of porous structures.

The cavity can be visualized using any chemical visualization software, as the cavity output is stored in a PDB file containing the cavity grid points. To facilitate the use of the algorithm for nonspecialized users, a plugin for the molecular viewer PyMol was developed, enabling its use without requiring computer programming knowledge. We anticipate that the developed software will streamline the characterization of molecular cages and speed up the development of novel functional designs.

## Data Availability

Source code and associated Python files are freely available at https://github.com/VicenteMartiCentelles/CageCavityCalc

## Supporting Information Available

Description of the arguments that can be used in the *C*3 Python module through the command line and in Python scripts, description of the *C*3 PyMol plugin GUI use, installation instructions, parameters, and details of the calculated cavities (PDF)
*C*3 source code v1.0.5 (ZIP)
Tutorial of the PyMol plugin (MP4)
*C*3 computed cavity volume for the MD trajectory of cage **C10** (MP4)
