# Supplementary material for: CageCavityCalc (C3): A Computational Tool for Calculating and Visualizing Cavities in Molecular Cages
Source: J Chem Inf Model. 2024 Jul 9;64(14):5604–16. doi: 10.1021/acs.jcim.4c00355 (PMC11267575; doi:10.1021/acs.jcim.4c00355)
Supplement: Supplementary file 1 — ci4c00355_si_001.pdf [file ci4c00355_si_001.pdf]

Supporting Information

CageCavityCalc (*C3*): A computational tool for calculating and visualizing cavities in Molecular Cages

Vicente Martí-Centelles, *[a,b,c] *Tomasz K. Piskorz,*[d] *Fernanda Duarte,* *[d]

[a] Instituto Interuniversitario de Investigación de Reconocimiento Molecular y Desarrollo Tecnológico (IDM), Universitat Politècnica de València, Universitat de València, Valencia 46022, Spain

[b] CIBER de Bioingeniería Biomateriales y Nanomedicina, Instituto de Salud Carlos III, Spain

[c] Departamento de Química, Universitat Politècnica de València, Camí de Vera s/n, 46022, Valencia, Spain

[d] Chemistry Research Laboratory, University of Oxford, Mansfield Road, Oxford OX1 3TA, United Kingdom

Contents

S1. Python module and command line interface 3
S2. PyMol plugin 7
S3. Installation instructions 9
S4. Examples of cavity calculation for cages **C1–C16** 11
S5. Evaluation of the Protein-Orientation Sensitivity (POS) 15
S6. Evaluation of the Grid-Spacing Sensitivity (GSS) 18
S7. Evaluation of the Mouth Opening Ambiguity (MOA) 19
S8. Systematic comparison with other cavity calculation software 20
S9. Evaluation performance against cavity volume estimates obtained from Rebek's 55% rule . 25

S1. Python module and command line interface

The module can be used from the command line or from a python file by loading the CageCavityCalc module. To use *C3* from the command line the user needs to execute in the Anaconda Prompt the following commands: *$CageCavityCalc -f cage.pdb -o cage_cavity.pdb -gr 1.5*. This order will load the cage.pdb file containing the cage chemical structure and the cavity of the cage will be calculated using a grid spacing of 1.5 Å. Additional arguments can be used as described in Table S1, allowing specifying the distance threshold used to calculate 90º angle, the use of the clustering algorithm to remove noisy cavity points that do not belong to the main cavity, calculation of hydrophobicity specifying the method and distance function, calculation of ESP, save a PyMol pml file, or print additional information of the calculations in the terminal, etc..

Table S1. Arguments that can be used in the *C3* Python module though the command line.

Argument	Action
-f	Input file (*pdb, *mol2, ...)
-o	Output file (*pdb, *mol2, ...). If this argument is not used, the automatic generation of output filenames is performed.
-gr *X*	Grid spacing resolution (Angstroms). Default 1.0 Å
-d90a *X*	Automatic distance threshold to calculate 90 deg angle as *X* times window radius. Default 2.0. If the calculated threshold distance is smaller than 5 Å, it is set to 5 Å to ensure probe to find atoms to calculate the angle.
-d90m *X*	Manual distance threshold to calculate 90 deg angle in Å. Default value is set to 5 Å.
-cluster *false*, *size* or *dist*	Remove cavity noise by dbscan clustering (size or dist)
-hydrophobicity or -hydro	Calculate hydrophobicity
-method *Method*	Method to calculate the hydrophobicity: Ghose or Crippen
-distfun *Function*	Method to calculate the hydrophobicity: Audry, Fauchere, Fauchere2, OnlyValues
-esp	Calculate the electrostatic potential
-charge_method M*ethod*	Charge method used in the ESP: eem, mmff94, gasteiger, qeq, qtpie, eem2015ha, eem2015hm, eem2015hn, eem2015ba, eem2015bm, eem2015bn. Default=eem.
-metal *M*	Metal used in the ESP
-metal_charge *X*	Charge of the metal used in the ESP
-pymol	Create PyMol pml file
-info	Print log INFO on the terminal

To use *C3* as in a Python script, it is required to load the module, followed by the initialization of the cavity, load the .pdb file of the cage, followed by the cavity volume calculation (using the default values of grid spacing resolution 1 Å and distance threshold for the 90-degree calculation of 5 Å) and saving the corresponding *.pdb file and PyMol *.pml file for cavity visualization in PyMol.

```
from CageCavityCalc.CageCavityCalc import cavity
cav = cavity()
cav.read_file("cage.pdb")
volume = cav.calculate_volume()
cav.print_to_file("cage_cavity.pdb")
cav.print_to_pymol("cage_cavity.pml")
print("Cavity_volume= ", volume, " A3")
```

We also provide a more complex example to show additional functionality of *C3*. In the example below it is loaded the cage.pdb file, then the cavity is computed using a grid spacing of 1.0 Å and a distance threshold for the 90-degree calculation of 2.0 times the window size. Note that this code uses the same implementation of the distance threshold for the 90-degree of the PyMol plugin. If computed window size is very small, resulting in threshold for the 90-degree smaller than 5 Å, the threshold is set to 5 Å to ensure the probe to find atoms to calculate the angle. The cavity is be saved into *.pdb file and also a PyMol *.pml file to facilitate cavity visualization in PyMol. The calculated properties can also be saved, the example below shows how to calculate the hydrophobicity and ESP and save individual *.pdb files with the hydrophobicity and ESP values stored in the B-factor, as well as PyMol *.pml files to facilitate cavity visualization with the properties in PyMol.

```
from CageCavityCalc.CageCavityCalc import cavity

cage_name = "cage"
grid_spacing = 1.0
distance_threshold_for_90_deg_angle = 2.0

cav = cavity()
```

```
cav.read_file(cage_name+".pdb")
window_radius = cav.calculate_window()
cav.distance_threshold_for_90_deg_angle = window_radius * distance_threshold_for_90_deg_angle
if cav.distance_threshold_for_90_deg_angle < 5:
    cav.distance_threshold_for_90_deg_angle = 5
cav.grid_spacing = float(grid_spacing)
cav.dummy_atom_radii = float(grid_spacing)
volume = cav.calculate_volume()
cav.print_to_file(cage_name+"_cavity.pdb")
cav.print_to_pymol(cage_name+"_cavity.pml")
cav.hydrophMethod = "Ghose" #Ghose or Crippen
cav.distance_function = "Fauchere" # Audry, Fauchere, Fauchere2, OnlyValues
cav.calculate_hydrophobicity()
cav.print_to_file(cage_name+"_cavity_hydrophobicity.pdb")
cav.print_to_pymol(cage_name+"_cavity_hydrophobicity.pml", 'h')
cav.calculate_esp() #If metals: cav.calculate_esp(metal_name="Pd", metal_charge=2)
cav.print_to_file(cage_name+"_cavity_esp.pdb")
cav.print_to_pymol(cage_name+"_cavity_esp.pml", "esp")
print("Cavity_volume= ", volume, " A3")
```

The Python module can be integrated into more complex programs, for example, it can be used to read a cage class from Cgbind, that enables the cage construction from a ligand smile structure, a metal, and the cage topology:

```
from CageCavityCalc.CageCavityCalc import cavity
from cgbind import Linker, Cage

linker = Linker(smiles='C1(C#CC2=CC=CC(C#CC3=CC=CN=C3)=C2)=CC=CN=C1', arch_name='m2l4')
cage = Cage(linker, metal='Pd')

cav = cavity()
cav.read_cgbind(cage)
cav.calculate_volume()
cav.print_to_file("cage_cavity.pdb")
```

It can also be used for reading MDAnalysis universe files:

```
from CageCavityCalc.CageCavityCalc import cavity
import MDAnalysis

cav = cavity()

syst = MDAnalysis.Universe("short.gro", "short.xtc")

volume = []
for ts in syst.trajectory:
    cav.read_mdanalysis(syst)
    volume.append(cav.calculate_volume())

print(volume)
```

The obtained cavity volumes in all the structures of the trajectory can be saved using the following script. The number of atoms is kept constant by setting additional grid points is in existing grid point, or in case when there is no cavity it is set to (0,0,0), when necessary.

```
from CageCavityCalc.CageCavityCalc import cavity
import MDAnalysis
import numpy as np


syst = MDAnalysis.Universe("short.gro", "short.xtc")
volume = []
max_grid = 0

for idx, ts in enumerate(syst.trajectory):
    cav = cavity()
    cav.read_mdanalysis(syst.atoms)
    volume.append(cav.calculate_volume())
    cav.print_to_file(f"cage_cavity_{idx:}.pdb")
    if len(cav.dummy_atoms_positions) > max_grid:
        max_grid = len(cav.dummy_atoms_positions)

atom_max = max_grid + cav.n_atoms

# Save as a trajectory:
FileTraj = MDAnalysis.Writer("traj.xtc")
for idx, ts in enumerate(syst.trajectory):
    syst = MDAnalysis.Universe(f"cage_cavity_{idx:}.pdb")
    n_missing = atom_max - len(syst.atoms)

    if n_missing != 0:  # save at the same position as existing grid
        positions = np.array([syst.atoms.select_atoms("name D and resname CV")[0].position] * n_missing)
    else:  # if no cavity, save it at the 0,0,0
        positions = np.zeros((n_missing, 3))

    if n_missing > 0:
        sol = MDAnalysis.Universe.empty(n_missing, trajectory=True)
        sol.add_TopologyAttr('name', ['D'] * n_missing)
        sol.add_TopologyAttr('type', ['D'] * n_missing)
        sol.add_TopologyAttr('resname', ['CV'])
        sol.atoms.positions = positions
        FileTraj.write(MDAnalysis.Merge(syst.atoms, sol.atoms).atoms)
    else:
        FileTraj.write(syst.atoms)

FileTraj.close()
```

S2. PyMol plugin

The *C3* Python module is integrated into PyMol in a plugin. The plugin is integrated into the software through a user interface allowing the selection of the different parameters for the cavity calculation. First, the user needs to initiate PyMol by typing “pymol” in the Anaconda Prompt. Then, in the PyMol interface the user needs to load the desired cage file using File > Open and select the “cage.pdb” file. Then, to initiate the *C3* plugin, the user needs access to Plugin > CageCavityCalc. All the options that the user can adjust are described in Figure S1. Once all the options are selected, the user needs to click on “Calculate volume” to initiate the calculation of the cavity and all the selected properties. Once the computation is finished, the computed cavity and the cavity with the properties are displayed in PyMol. The PyMol plugin enables the storage of all computed properties in the same PyMol session file, allowing the user to select which one to display and to save PDB files of each property. To save the session file, the user needs to access to File > Save Session As. The user can select the computed property to display by just clicking on the right panel of the generated cavity objects, *i.e.* click over the cavity object to toggle between hide/show. (see Figure 9 in the manuscript). To obtain a good quality image of the cage and the cavity, the user needs to type “ray” in the PyMol command line, then the obtained image can be saved by using File > Export Image As > PNG.

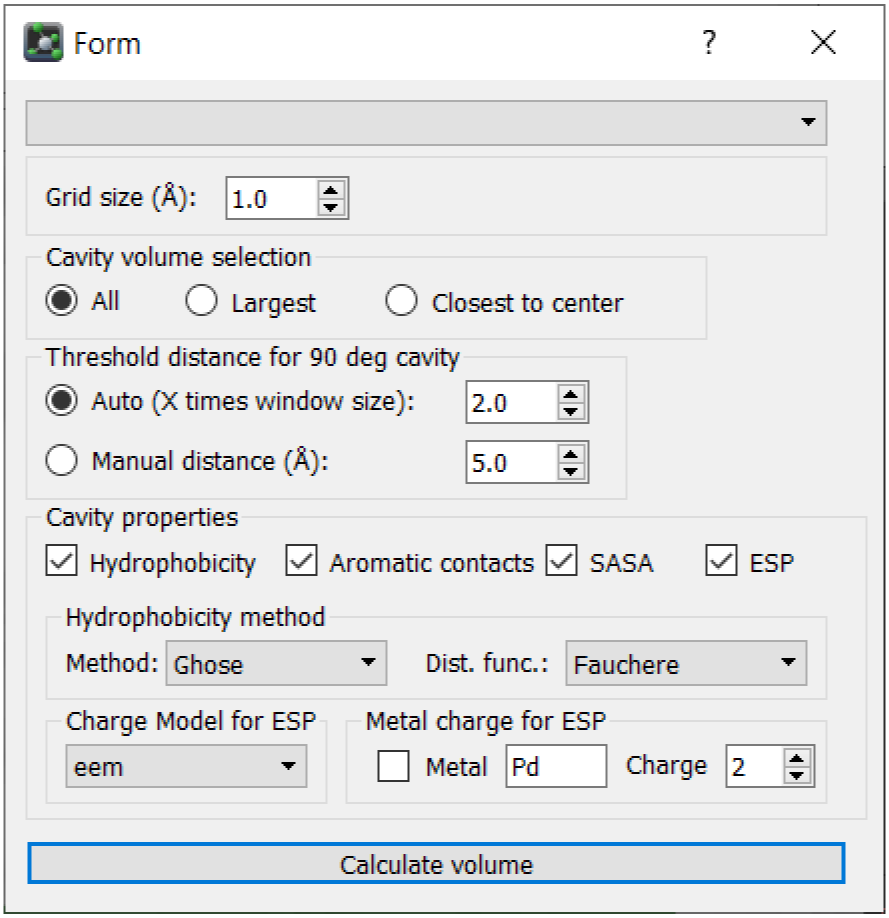

Figure S1. Screenshot of the *C3* GUI of the PyMol plugin.

S3. Installation instructions

The installation of *C3* requires the following steps. The software is compatible with Linux, Windows, and macOS.

First it is required to install Miniconda3 (Python 3.7 or later,), that can be obtained from https://docs.conda.io/en/latest/miniconda.html. Then in the "Anaconda Prompt", the command line version of *C3* is installed using pip: "pip install CageCavityCalc", this will install the required dependencies.

For performing ESP and hydrophobicity calculation, *C3* requires OpenBabel that can be installed using "conda install -c conda-forge openbabel" (or "conda config --add channels conda-forge" followed by "conda install openbabel"). For file loading in multiple formats, including files for molecular dynamics cavity analysis, requires installing MDAnalysis using "conda install -c conda-forge mdanalysis" (or "conda config --add channels conda-forge" followed by "conda install mdanalysis").

As with any program, to run CageCavityCalc from the command line it is needed to either add its installation folder to the system path or to execute the CageCavityCalc.py file directly from the folder. For example, in Windows the user needs to add the folder "C:\Users\UserName\miniconda3\Lib\site-packages\CageCavityCalc\" to the Python path navigating through the following menus: My Computer > Properties > Advanced System Settings > Environment Variables > PYTHONPATH.

To install the *C3* PyMol plugin, the open-source version of PyMol must be installed in the "Anaconda Prompt" the command using "conda install -c conda-forge pymol-open-source" for Windows, macOS, and Linux. Alternatively, it can be installed from https://www.cgohlke.com/ for Windows. It is also required to install the following dependencies: "pip install pyqt5 qtpy" (in

some cases it may require uninstall pyqt5 with “pip uninstall pyqt5” followed by “pip install pyqt5 qtpy”). For Spanish computers, for running the plugin it is required to change the regional settings of the computer to use points as a decimal separator instead of commas.

Once PyMol is installed, in PyMol the plugin is installed from: Plugin > Plugin Manager > Install New Plugin. Choose “Install from local file” and locate the __init__.py file in the pymol_plugin folder of *C3* typically located in C:\Users\UserName\miniconda3\Lib\site-packages\CageCavityCalc\pymol_plugin. To use the plugin, the user just needs to open a cage in PyMol, then go to Plugin and click on CageCavityCalc to run the plugin. Then, the computed cavity is displayed in PyMol. The user can select the computed property to display by just clicking on the right panel of the generated cavity objects.

Summary of the required installation actions and commands

Download and install Miniconda3 (https://docs.conda.io/en/latest/miniconda.html)

Open the “Anaconda Prompt” and execute the following commands:

```
pip install CageCavityCalc
conda install -c conda-forge pymol-open-source
pip uninstall pyqt5
pip install pyqt5 qtpy
conda install -c conda-forge openbabel
conda install -c conda-forge mdanalysis
pymol
```

Then, install the PyMol the plugin: Plugin > Plugin Manager > Install New Plugin. Choose “Install from local file” and locate the __init__.py file in the pymol_plugin folder of *C3* typically located in C:\Users\UserName\miniconda3\Lib\site-packages\CageCavityCalc\pymol_plugin.

S4. Examples of cavity calculation for cages C1–C16

In our cavity calculations we used the X-ray diffraction structure of the cages from the Cambridge Structural Database (CSD). The cage structures were prepared from the original CIF files from the CSD by removing non-cage molecules using Wavefunction Spartan '20.[S1] Additionally, the outward-pointing phenyl groups of cage **C9** were removed.

The cavity of cages **C1–C16** were calculated using *C3* and the optimized parameters described in Table S2. For parameter optimization, the grid spacing for small-medium cages **C1–C12** (*i.e.* cavity volumes less than 1000 Å^3) to 0.5 Å. The larger cages **C13–C16** required increasing the grid spacing to 1.0–3.5 Å to run in a reasonable time and to adjust to hardware limitations. For each cage reported in Table S2, the overall calculation time takes from seconds to minutes (typically times vary from 30 seconds to 5 minutes) depending on grid resolution and cavity size in a PC computer with an Intel(R) Core(TM) i9-10900K CPU @ 3.70GHz processor.

[S1] Deppmeier, B. J.; Driessen, A. J.; Hehre, T. S.; Hehre, W. J.; Johnson, J. A.; Klunzinger, P. E.; Leonard, J. M.; Pham, I. N.; Pietro, W. J.; Jianguo, Y. Spartan '20, version 1.0.0 (Mar 8th 2021), Wavefunction Inc., 2011.

Table S2. Computed cavity volumes and parameters used in *C3* for cages **C1–C16**. [a] Distance threshold for the 90-degree calculation in window size units. The cavity volume obtained using the default parameters of grid spacing 1 Å (except for cages **C15** and **C16**, that hardware limitations required using larger grid spacing) and distance threshold 2 times the window size is reported in brackets. The mean relative absolute error (MRAE) of cavity volumes computed with the optimized parameters and the default parameters is 26.7%.

Cage	CCDC	Volume ($Å^3$)	Grid spacing (Å)	Distance threshold[a]	Computed cavity
**C1**	1970309	209<br>(116)	0.5<br>(1.0)	2.0<br>(2.0)	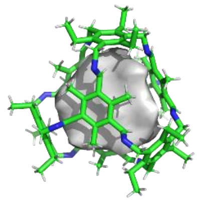
**C2**	718469	295<br>(190)	0.5<br>(1.0)	2.0<br>(2.0)	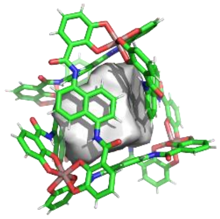
**C3**	1862753	63<br>(26)	0.5<br>(1.0)	2.0<br>(2.0)	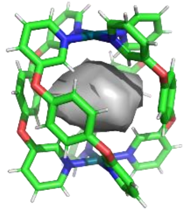
**C4**	1872778	726<br>(546)	0.5<br>(1.0)	2.0<br>(2.0)	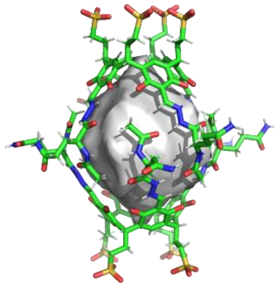
**C5**	2068666	699<br>(484)	0.5<br>(1.0)	2.0<br>(2.0)	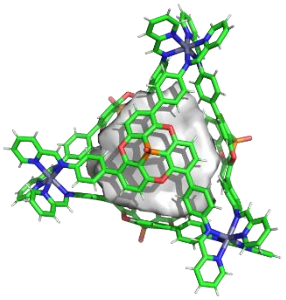

**C6**	1492902	308 (201)	0.5 (1.0)	2.0 (2.0)	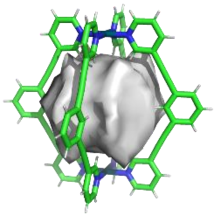
**C7**	2157968	496 (391)	0.5 (1.0)	2.0 (2.0)	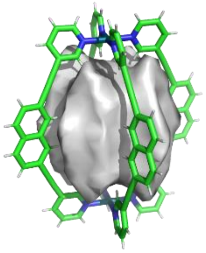
**C8**	2053043	130 (61)	0.5 (1.0)	2.0 (2.0)	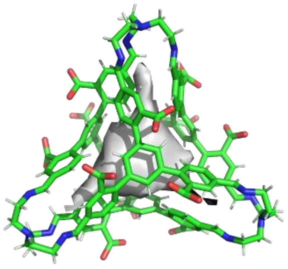
**C9**	1892128	672 (580)	0.5 (1.0)	2.0 (2.0)	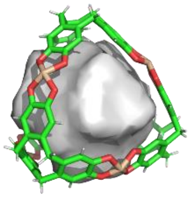
**C10**	794242	667 (527)	0.5 (1.0)	2.0 (2.0)	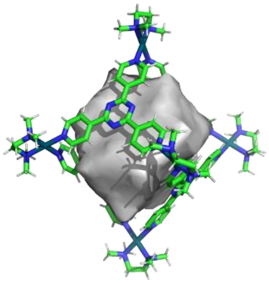
**C11**	1541839	458 (331)	0.5 (1.0)	2.0 (2.0)	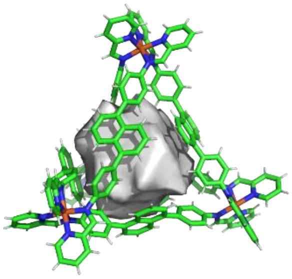

**C12**	1416694	613<br>(397)	0.5<br>(1.0)	2.0<br>(2.0)	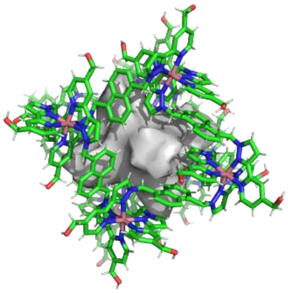
**C13**	2161290	5240<br>(6097)	1.0<br>(1.0)	3.0<br>(2.0)	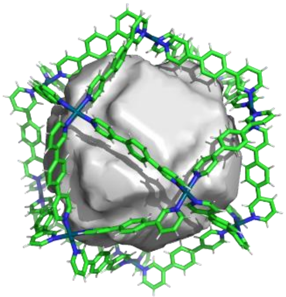
**C14**	2014846	7784<br>(8038)	1.0<br>(1.0)	3.0<br>(2.0)	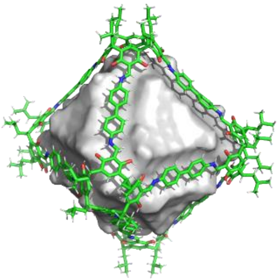
**C15**	1831430	31327<br>(31955)	3.0<br>(3.0)	4.5<br>(2.0)	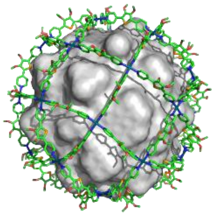
**C16**	1831431	65722<br>(67930)	3.5<br>(3.5)	4.5<br>(2.0)	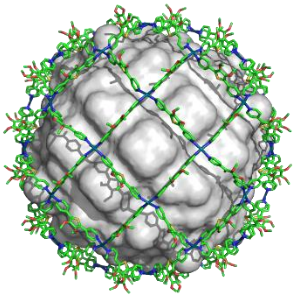

S5. Evaluation of the Protein-Orientation Sensitivity (POS)

The Protein-Orientation Sensitivity (POS) has been evaluated by randomly rotating the XYZ coordinates of cages **C1**–**C16** to obtain a total of 5 structures per cage (see Python code below). The volume of the cavity for each cage has been calculated using the parameters described in Table S3, and the mean volume and the standard error has been calculated. The error reported Table S3 is computed from the standard deviation of the 5 cavity volumes computed for each cage, *i.e.* the dispersion around the mean value.

Table S3. Evaluation of the Protein-Orientation Sensitivity (POS).

Cage	*C3* results
**C1**	V_{mean} = (210 ± 1) Å^3<br>Protein-Orientation Sensitivity (POS) = 0.3% error
**C2**	V_{mean} = (293 ± 2) Å^3<br>Protein-Orientation Sensitivity (POS) = 0.7% error
**C3**	V_{mean} = (65 ± 1) Å^3<br>Protein-Orientation Sensitivity (POS) = 0.9% error
**C4**	V_{mean} = (728 ± 2) Å^3<br>Protein-Orientation Sensitivity (POS) = 0.2% error
**C5**	V_{mean} = (698 ± 2) Å^3<br>Protein-Orientation Sensitivity (POS) = 0.4% error
**C6**	V_{mean} = (311 ± 2) Å^3<br>Protein-Orientation Sensitivity (POS) = 0.6% error
**C7**	V_{mean} = (493 ± 1) Å^3<br>Protein-Orientation Sensitivity (POS) = 0.3% error
**C8**	V_{mean} = (128 ± 1) Å^3<br>Protein-Orientation Sensitivity (POS) = 0.9% error
**C9**	V_{mean} = (650 ± 12) Å^3

	Protein-Orientation Sensitivity (POS) = 1.8 % error
**C10**	V_{mean} = (607 ± 17) $Å^3$<br>Protein-Orientation Sensitivity (POS) = 2.8 % error
**C11**	V_{mean} = (457 ± 1) $Å^3$<br>Protein-Orientation Sensitivity (POS) = 0.2 % error
**C12**	V_{mean} = (624 ± 4) $Å^3$<br>Protein-Orientation Sensitivity (POS) = 0.6 % error
**C13**	V_{mean} = (5299 ± 31) $Å^3$<br>Protein-Orientation Sensitivity (POS) = 0.6% error
**C14**	V_{mean} = (8010 ± 136) $Å^3$<br>Protein-Orientation Sensitivity (POS) = 1.7 % error
**C15**	V_{mean} = (29557 ± 634) $Å^3$<br>Protein-Orientation Sensitivity (POS) = 2.1 % error
**C16**	V_{mean} = (67198 ± 700) $Å^3$<br>Protein-Orientation Sensitivity (POS) = 1.1% error

Python code to randomly rotate the XYZ coordinates of the cage:

```
import numpy as np
import math as m

file_path = 'cage.xyz'

def Rx(theta):
  return np.matrix([[ 1, 0           , 0           ],
                   [ 0, m.cos(theta),-m.sin(theta)],
                   [ 0, m.sin(theta), m.cos(theta)]])

def Ry(theta):
  return np.matrix([[ m.cos(theta), 0, m.sin(theta)],
                   [ 0           , 1, 0           ],
                   [-m.sin(theta), 0, m.cos(theta)]])

def Rz(theta):
  return np.matrix([[ m.cos(theta), -m.sin(theta), 0 ],
                   [ m.sin(theta), m.cos(theta) , 0 ],
                   [ 0           , 0            , 1 ]])

def read_file_and_store_matrix(file_path):
    matrix = []
    with open(file_path, 'r') as file:
        for line in file:
            line = line.split()
```

```
            if line:
                matrix.append(line)
    return matrix

def iterate_matrix(matrix, R):
    out_matrix = []
    out_matrix.append(matrix[0])
    out_matrix.append(matrix[1])
    for row_idx in range(2, len(matrix)):
        row = matrix[row_idx]
        res = R*np.array([[float(row[1])],[float(row[2])],[float(row[3])]])
        out_matrix.append([row[0], res[0,0], res[1,0], res[2,0]])

    return out_matrix

def save_to_file(data, n, file_path):
    with open("out_"+ str(n) +"_"+file_path, 'w') as file:
        for row_idx in range(0, len(data)):
            row = data[row_idx]
            for element in row:
                file.write(str(element) + '\t')
            file.write('\n')
    file.close()

result_matrix = read_file_and_store_matrix(file_path)

for i in range(1, 5):
    print("## Rotation number " + str(i))
    phi = np.random.uniform(0, 2*np.pi)
    theta = np.random.uniform(0, 2*np.pi)
    psi = np.random.uniform(0, 2*np.pi)
    print("phi =", phi)
    print("theta  =", theta)
    print("psi =", psi)

    R = Rz(psi) * Ry(theta) * Rx(phi)

    transformed_matrix = iterate_matrix(result_matrix, R)

    save_to_file(transformed_matrix, i, file_path)
```

S6. Evaluation of the Grid-Spacing Sensitivity (GSS)

The Grid-Spacing Sensitivity (GSS) has been evaluated by increasing the grid-spacing from the parameters described in Table S2 for cages **C1–C16**.

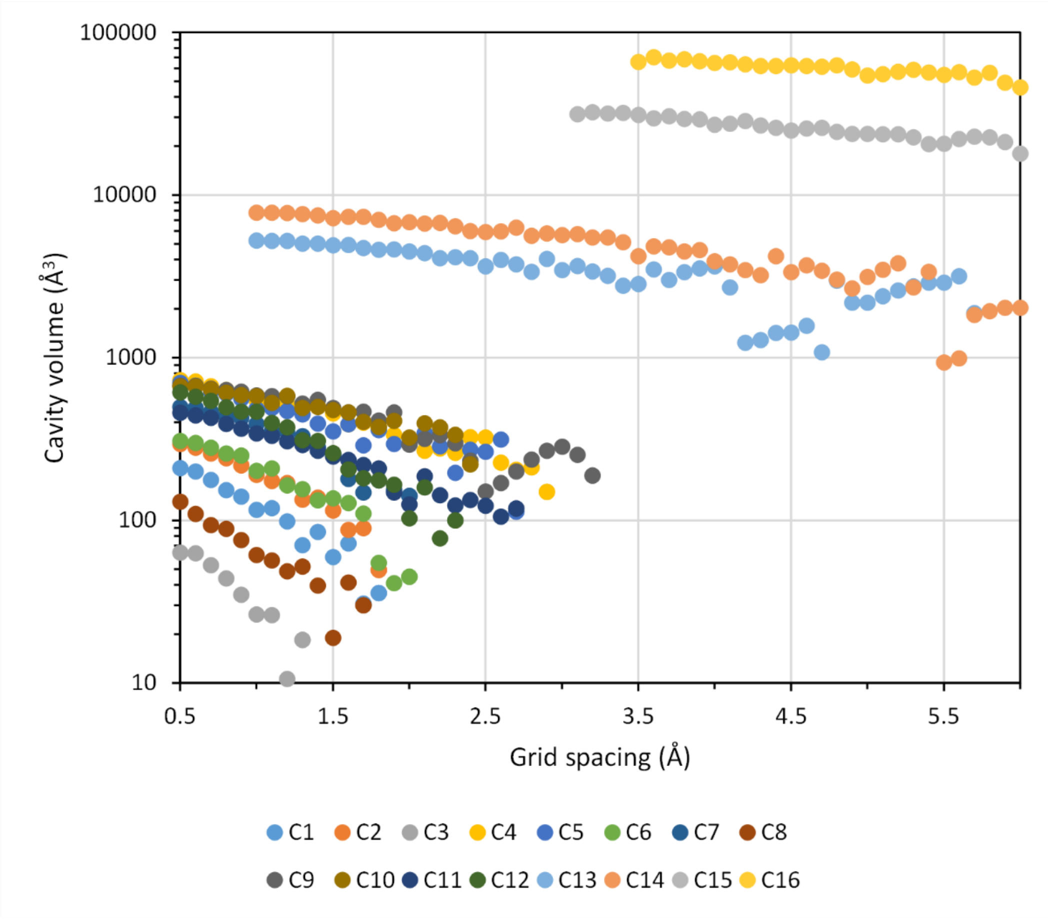

Figure S2. Evaluation of the Grid-Spacing Sensitivity (GSS) of *C3* for cages **C1–C16**.

S7. Evaluation of the Mouth Opening Ambiguity (MOA)

The Mouth Opening Ambiguity (MOA) has been evaluated by using distance threshold for the 90-degree calculation (dt_{90}) values from 1 to 5 in window size units, and the parameters described in Table S2. Note that if computed window size is very small, resulting in threshold for the 90-degree smaller than 5 Å, the threshold is set to 5 Å to ensure the probe to find atoms to calculate the angle. The cavity volume obtained for cages with small windows (**C1–C5**, **C8**, and **C11**) does not show any dependency on the distance threshold for the 90-degree calculation, and therefore it is not required any optimization by the user. Cages with larger windows have variable behaviors depending on the structure of each cage. Cages **C9**, **C11**, and **C12** do not show any dependency on dt_{90}, cage **C6** shows a 2% variation of the calculated volume from dt_{90} 1.0 to 2.5; if dt_{90} is more than 3.0, variations of the calculated volume up to –72% are observed. In contrast, the similarly shaped cage **C7** shows a 2% variation of the calculated volume over the whole test range dt_{90} 1.0 to 5.0. For cages with larger openings (**C13–16**), it is required to analyze visually the computed cavity and adjust the distance threshold for the 90-degree calculation. The obtained results are presented in Figure S3.

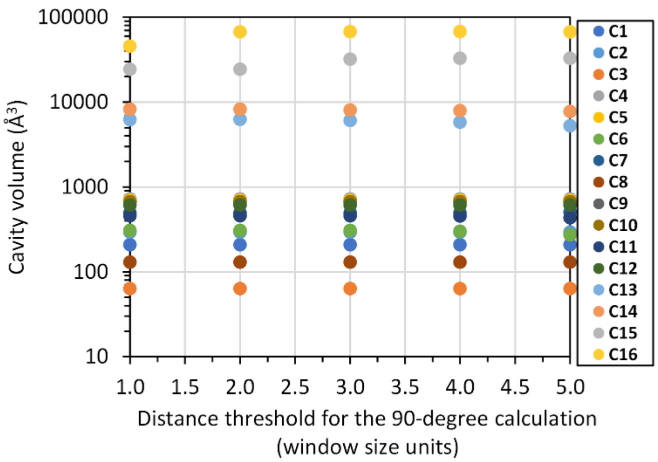

Figure S3. Evaluation of the Mouth Opening Ambiguity (MOA) of *C3* for cages **C1–C16.**.

S8. Systematic comparison with other cavity calculation software

We have performed a systematic comparison with different cavity calculation software (*i.e.* KVFinder, Fpocket, MoloVol, CAVER, ghecom, PyWindow, POVME, and MOLOVOL). For this, we tested the software already reviewed for calculating cavities for supramolecular cages from *J. Chem. Inf. Model.* **2023**, *63*, 3772.[S2] The parameters used for each software are presented in the following tables. All parameters not included in the tables were kept at their default values. Note that the program PyWindow has no customizable parameters, therefore there is no need to adjust any parameter.

Table S4. KVFinder project detection parameters (web version of parKVFinder version 1.2.0, https://kvfinder-web.cnpem.br/, accessed 20-04-2024).

Cage	CCDC	Step (Å)	Probe Out (Å)	Removal Distance (Å)	Volume Cutoff (Å^3)
**C1**	1970309	0.25	10	1.5	5
**C6**	1492902	0.25	20	1.5	5
**C7**	2157968	0.25	20	1.5	5
**C8**	2053043	0.25	5	1.5	5
**C10**	794242	0.25	15	1.5	5
**C13**	2161290	0.25	20	1.5	5
**C14**	2014846	0.25	20	1.5	5
**C16**	1831431	5.0	30	5.0	5

[S2] Guerra, J. V. S.; Alves, L. F. G.; Bourissou, D.; Lopes-De-Oliveira, P. S.; Szalóki, G. Cavity Characterization in Supramolecular Cages. *J. Chem. Inf. Model.* **2023**, *63*, 3772–3785. https://doi.org/10.1021/acs.jcim.3c00328.

Table S5. Fpocket detection parameters (FPocketWeb version 1.0.1, https://durrantlab.pitt.edu/fpocketweb/, accessed 20/4/2024).

Cage	CCDC	Minimum radius of an alpha sphere (Å)	Maximum radius of an alpha sphere (Å)
**C1**	1970309	3.4	6.2
**C6**	1492902	3.4	40
**C7**	2157968	3.4	40
**C8**	2053043	3.4	8.0
**C10**	794242	3.4	8.0
**C13**	2161290	3.4	40
**C14**	2014846	3.4	40
**C16**	1831431	4.0	40

Table S6. MoloVol detection parameters (desktop version 1.0.0, https://molovol.com/, accessed 20/4/24).

Cage	CCDC	Grid spacing (Å)	Small probe radius (Å)	Large probe radius (Å)
**C1**	1970309	0.6	1.4	5.0
**C6**	1492902	0.6	1.4	5.0
**C7**	2157968	0.6	1.4	5.0
**C8**	2053043	0.6	1.4	5.0
**C10**	794242	0.6	1.4	5.0
**C13**	2161290	1.0	1.4	10
**C14**	2014846	0.6	1.4	12
**C16**	1831431	2.0	1.4	25

Table S7. CAVER project detection parameters (CAVER Analyst 2.0 desktop version, installer downloaded from https://caver.cz/, accessed 20/4/24).

Cage	CCDC	Small probe radius (Å)	Larger probe radius (Å)
**C1**	1970309	1.4	3.0
**C6**	1492902	1.4	5.0
**C7**	2157968	1.4	5.0
**C8**	2053043	1.4	3.0
**C10**	794242	1.4	5.0
**C13**	2161290	1.4	7.0
**C14**	2014846	1.4	10.0
**C16**	1831431	1.4	8.0

Table S8. Ghecom detection parameters (Web version, https://pdbj.org/ghecom/, accessed 20/4/24).

Cage	CCDC	Maximium large probe shpere radius (Å)
**C1**	1970309	10.0
**C6**	1492902	4.0
**C7**	2157968	4.0
**C8**	2053043	10.0
**C10**	794242	10.0
**C13**	2161290	12.0
**C14**	2014846	14.0
**C16**	1831431	20.0

For POVME, the center of the inclusion region was set to center of mass. The radius was chosen visually to best represent the cavity.

Table S9. POVME detection parameters (Python version 3.0, https://github.com/POVME/POVME3, accessed 20/4/24).

Cage	CCDC	Inclusion Region
**C1**	1970309	Sphere centered at [19.98,6.22,12.43] with radius 9.3 Å
**C6**	1492902	Sphere centered at [0.26,17.55,12.52] with radius 6.0 Å
**C7**	2157968	Sphere centered at [-9.32,0.34,45.34] with radius 7.0 Å
**C8**	2053043	Sphere centered at [15.85,9.96,27.12] with radius 5.5 Å
**C10**	794242	Sphere centered at [13.26,-18.60,16.06] with radius 13.01 Å
**C13**	2161290	Sphere centered at [27.48,71.95,29.49] with radius 12.0 Å
**C14**	2014846	Sphere centered at [0.00,0.00,26.17] with radius 14.0 Å
**C16**	1831431	Sphere centered at [16.93,57.10,62.70] with radius 30.0 Å (with grid spacing 4 Å)

For VOIDOO calculations, the radius of the probe was chosen to be the smallest radius at which the probe does not escape the cavity.

Table S10. VOIDOO detection parameters (version 3.1.3, https://www.esrf.fr/exp_facilities/px_soft/usf/voidoo_man.html, accessed 20/4/24).

Cage	CCDC	Probe radius (Å)
**C1**	1970309	1.0
**C2**	718469	1.0
**C3**	1862753	1.0
**C4**	1872778	1.0
**C5**	2068666	1.2
**C6**	1492902	2.2
**C7**	2157968	2.4
**C8**	2053043	1.0
**C9**	1892128	3.0
**C10**	794242	3.0
**C11**	1541839	1.8
**C12**	1416694	1.8
**C13**	2161290	4.8
**C14**	2014846	4.8
**C15**	1831430	5.4
**C16**	1831431	5.2

S9. Evaluation performance against cavity volume estimates obtained from Rebek's 55% rule

In order to compare the performance of *C3* against cavity volume estimates, we used the cavity volume obtained from the Rebek's 55% rule for the inclusion complexes of the of Benchmark Dataset 1 reported in *J. Chem. Inf. Model.* **2023**, *63*, 3772.[S2] The accuracy of the volume estimation using mean relative absolute error (MRAE) is described in Table S11.

Table S11. Cavity volumes calculated with *C3* using a grid spacing of 0.5 Å and a distance threshold for the 90-degree calculation of 2 times the window size. for the Benchmark Dataset 1 from *J. Chem. Inf. Model.* **2023**, *63*, 3772.[S2] (Cage files downloaded from https://github.com/LBC-LNBio/SMC-Benchmarking/tree/main/hosts, accessed 23/5/24).

Cage	*C3* Calculated Volume (Å^3)	Guest vdW Volume (Å^3)	Estimated cavity volume using Rebek's rule (Å^3)	Relative Error (%)
**B1**	298	150	273	9.2
**B2**	292	155	281	3.8
**B3**	267	137	248	7.7
**B4**	422	309	562	-24.9
**B5**	63	50	90	-29.8
**B6**	64	53	96	-33.4
**B7**	749	519	944	-20.6
**B8**	726	512	930	-21.9
**B9**	85	141	257	-67.1
**B10**	263	151	274	-4.1
**B11**	448	307	558	-19.8
**B12**	708	524	954	-25.8
**B13**	828	618	1123	-26.2
			**MRAE (%)**	22.6
