# Supplementary figures and images for: CageCavityCalc (C3): A Computational Tool for Calculating and Visualizing Cavities in Molecular Cages
Source: J Chem Inf Model. 2024 Jul 9;64(14):5604–16. doi: 10.1021/acs.jcim.4c00355 (PMC11267575; doi:10.1021/acs.jcim.4c00355)

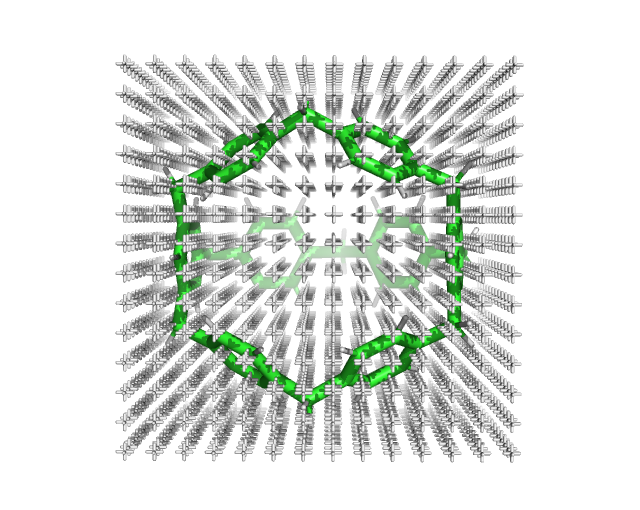

Supplement: Supplementary file 2 — ci4c00355_si_002.zip [file ci4c00355_si_002.zip › CageCavityCalc-v.1.0.5/CageCavityCalc/examples/12_test_cage/fig_1.png]

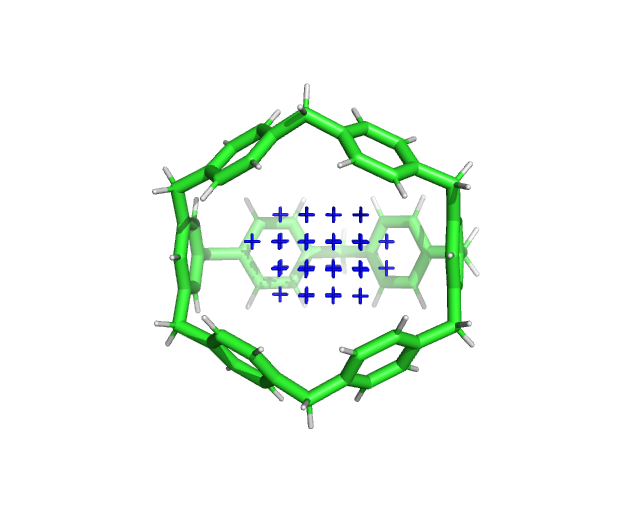

Supplement: Supplementary file 2 — ci4c00355_si_002.zip [file ci4c00355_si_002.zip › CageCavityCalc-v.1.0.5/CageCavityCalc/examples/12_test_cage/fig_2.png]

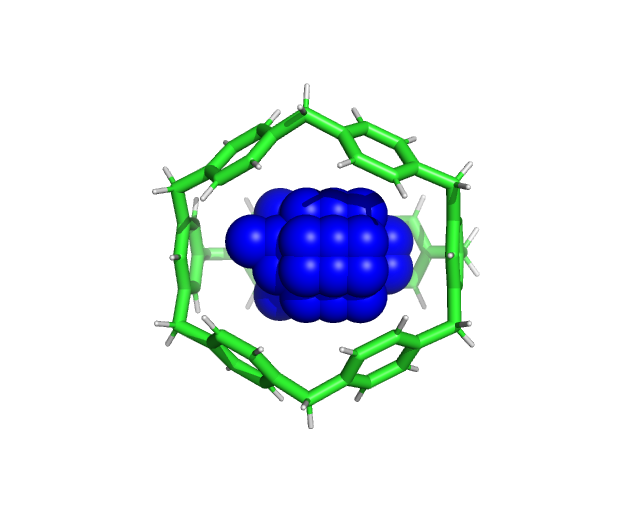

Supplement: Supplementary file 2 — ci4c00355_si_002.zip [file ci4c00355_si_002.zip › CageCavityCalc-v.1.0.5/CageCavityCalc/examples/12_test_cage/fig_3.png]

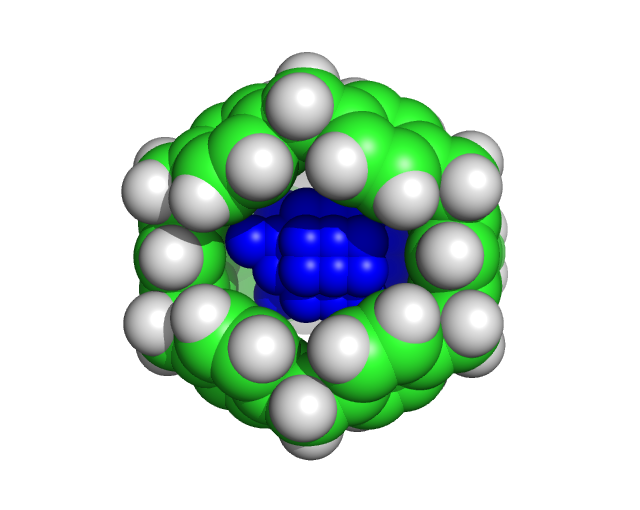

Supplement: Supplementary file 2 — ci4c00355_si_002.zip [file ci4c00355_si_002.zip › CageCavityCalc-v.1.0.5/CageCavityCalc/examples/12_test_cage/fig_4.png]

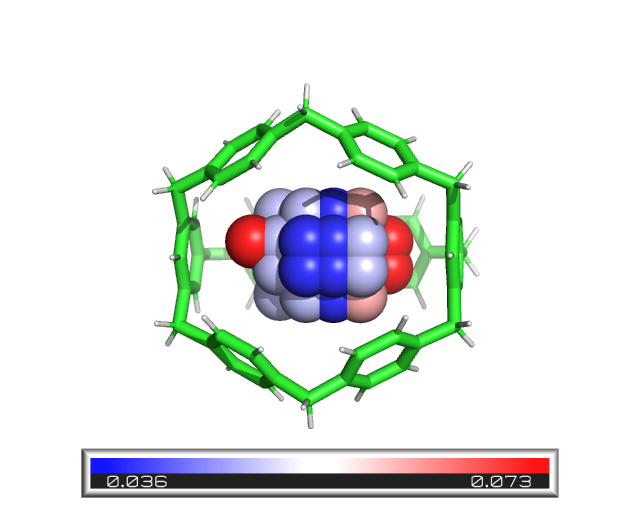

Supplement: Supplementary file 2 — ci4c00355_si_002.zip [file ci4c00355_si_002.zip › CageCavityCalc-v.1.0.5/CageCavityCalc/examples/12_test_cage/fig_5.png]

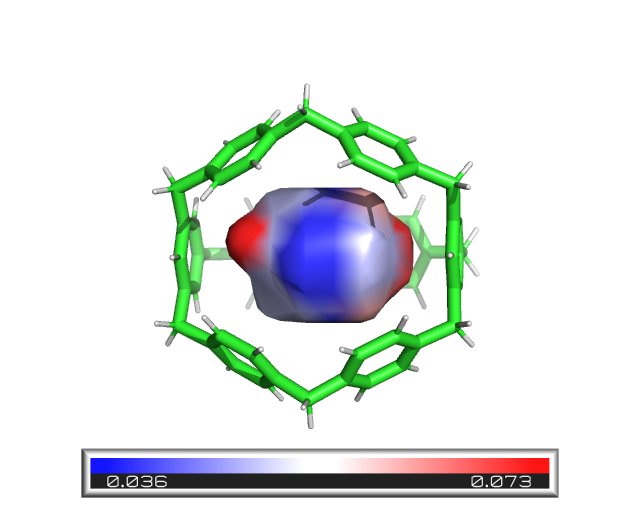

Supplement: Supplementary file 2 — ci4c00355_si_002.zip [file ci4c00355_si_002.zip › CageCavityCalc-v.1.0.5/CageCavityCalc/examples/12_test_cage/fig_6.png]

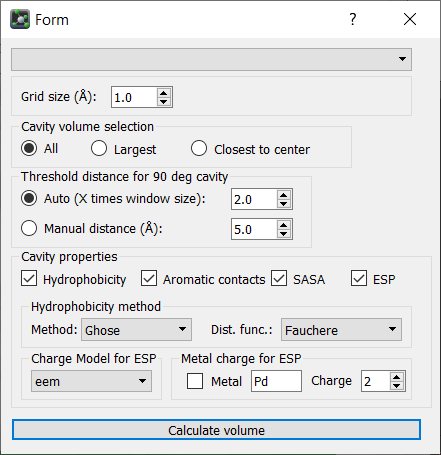

Supplement: Supplementary file 2 — ci4c00355_si_002.zip [file ci4c00355_si_002.zip › CageCavityCalc-v.1.0.5/CageCavityCalc/pic/C3_PyMol_Plugin.png]

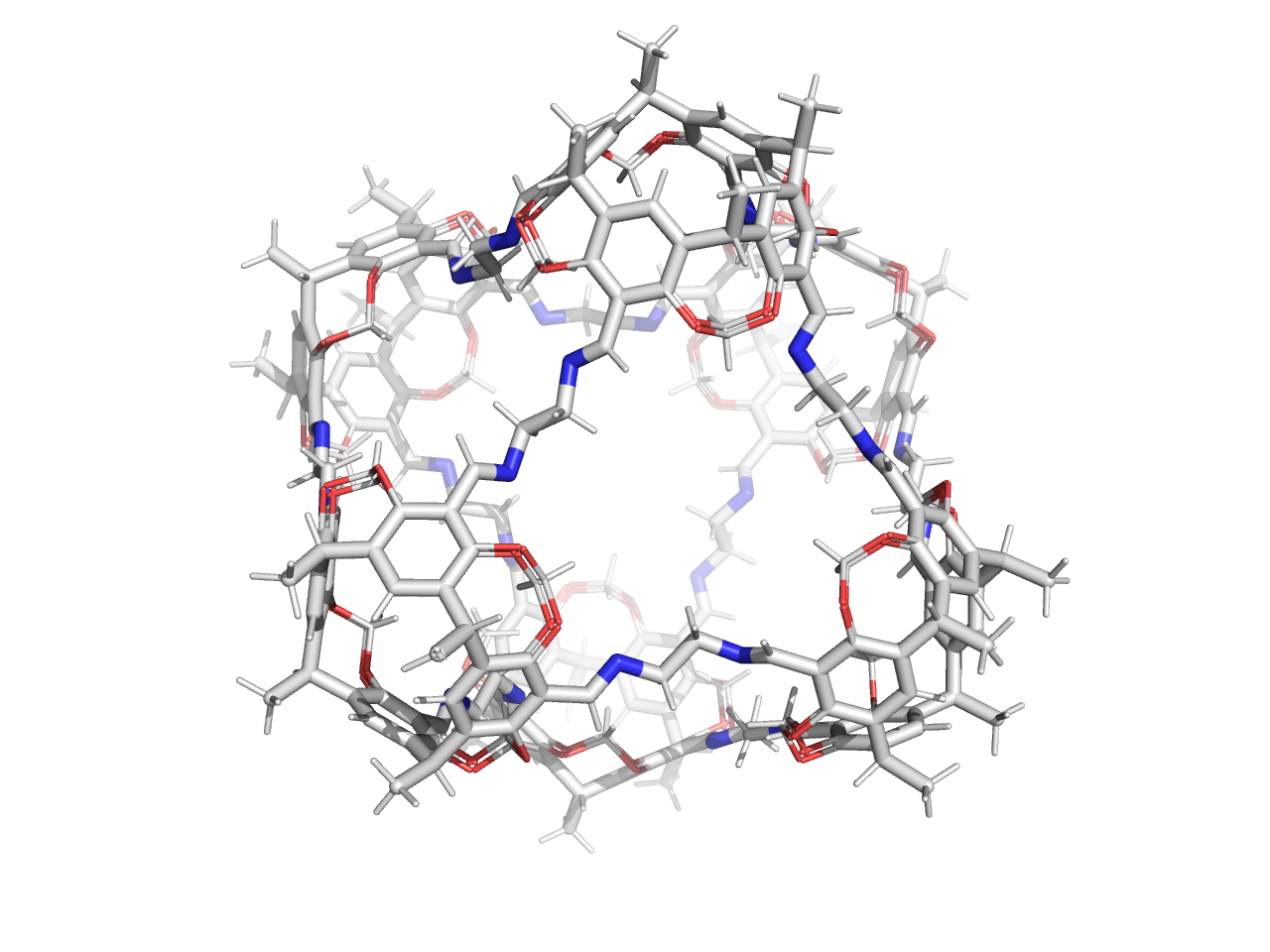

Supplement: Supplementary file 2 — ci4c00355_si_002.zip [file ci4c00355_si_002.zip › CageCavityCalc-v.1.0.5/CageCavityCalc/pic/cage.png]

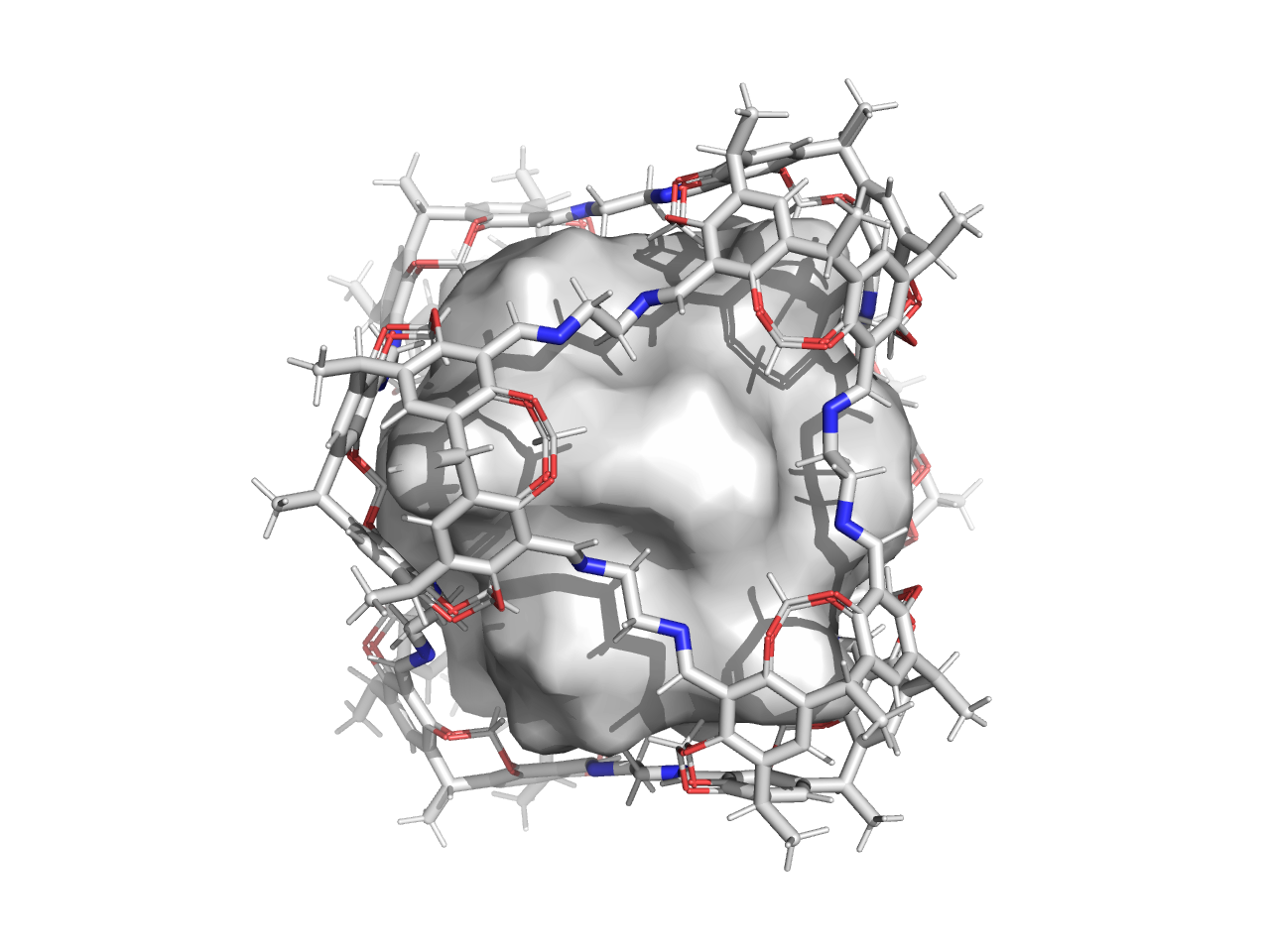

Supplement: Supplementary file 2 — ci4c00355_si_002.zip [file ci4c00355_si_002.zip › CageCavityCalc-v.1.0.5/CageCavityCalc/pic/cavity.png]

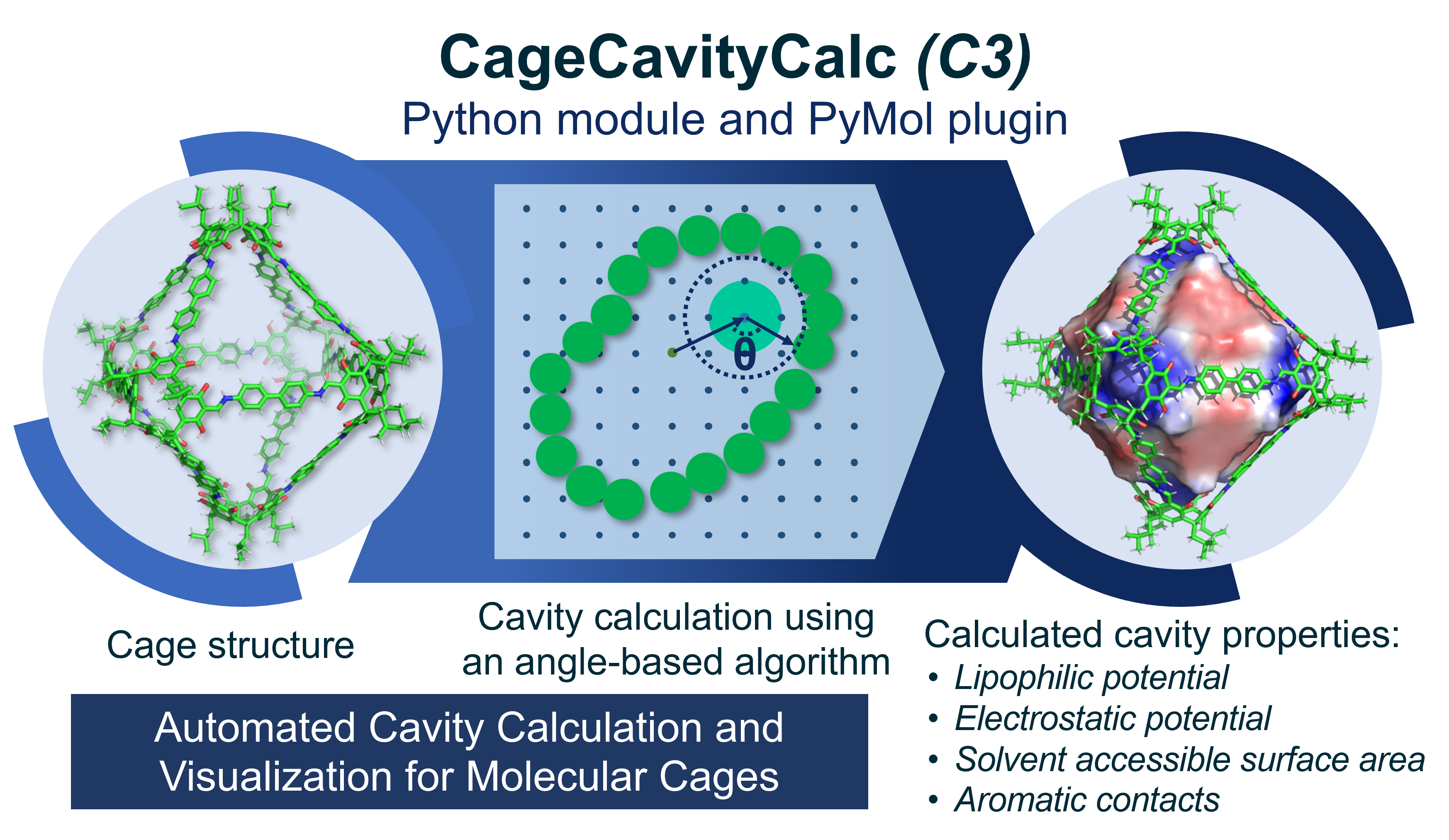

Supplement: Supplementary file 2 — ci4c00355_si_002.zip [file ci4c00355_si_002.zip › CageCavityCalc-v.1.0.5/CageCavityCalc/pic/graphical_abstract_CageCavityCalc.png]

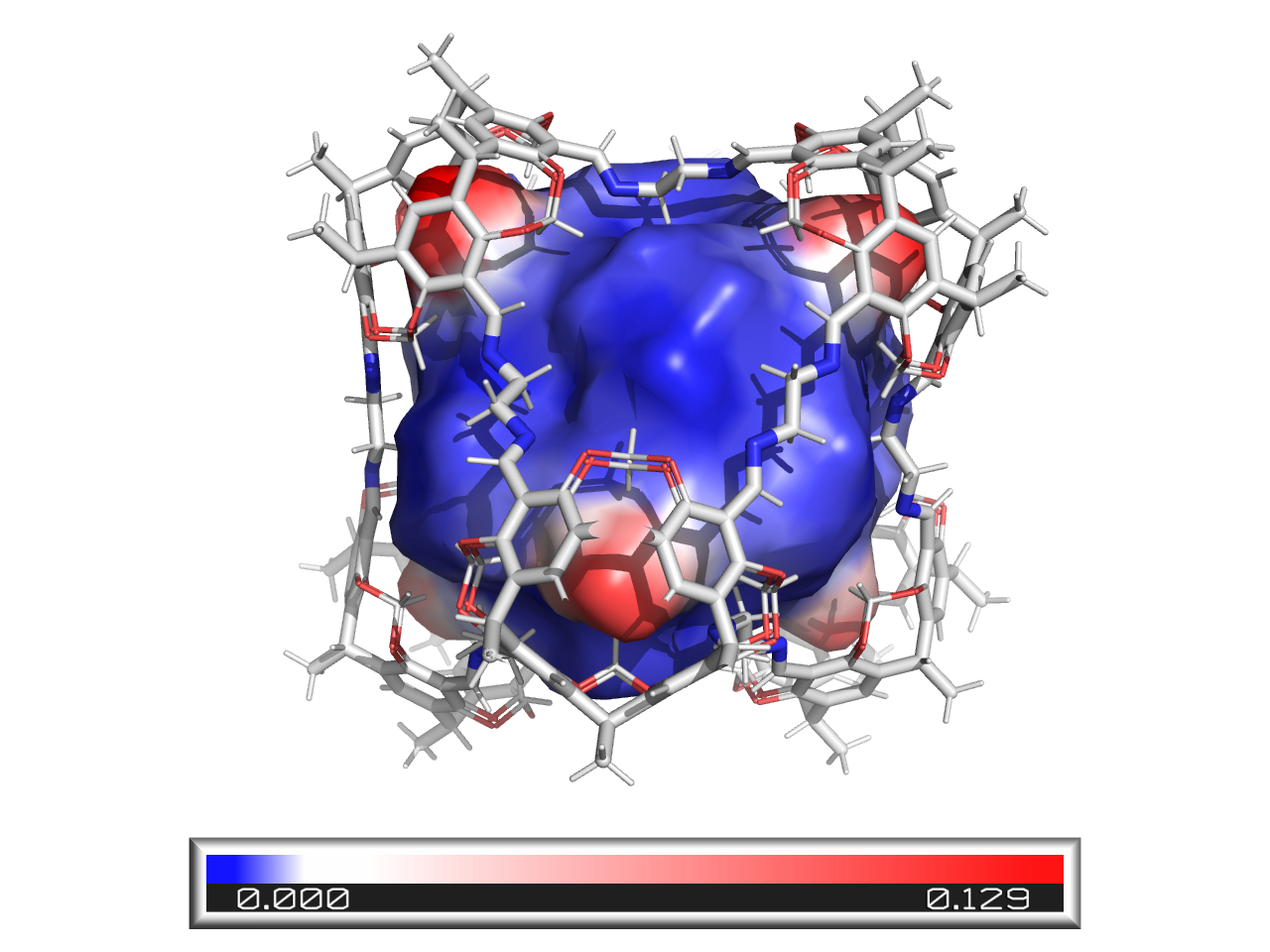

Supplement: Supplementary file 2 — ci4c00355_si_002.zip [file ci4c00355_si_002.zip › CageCavityCalc-v.1.0.5/CageCavityCalc/pic/hydrophobicity.png]

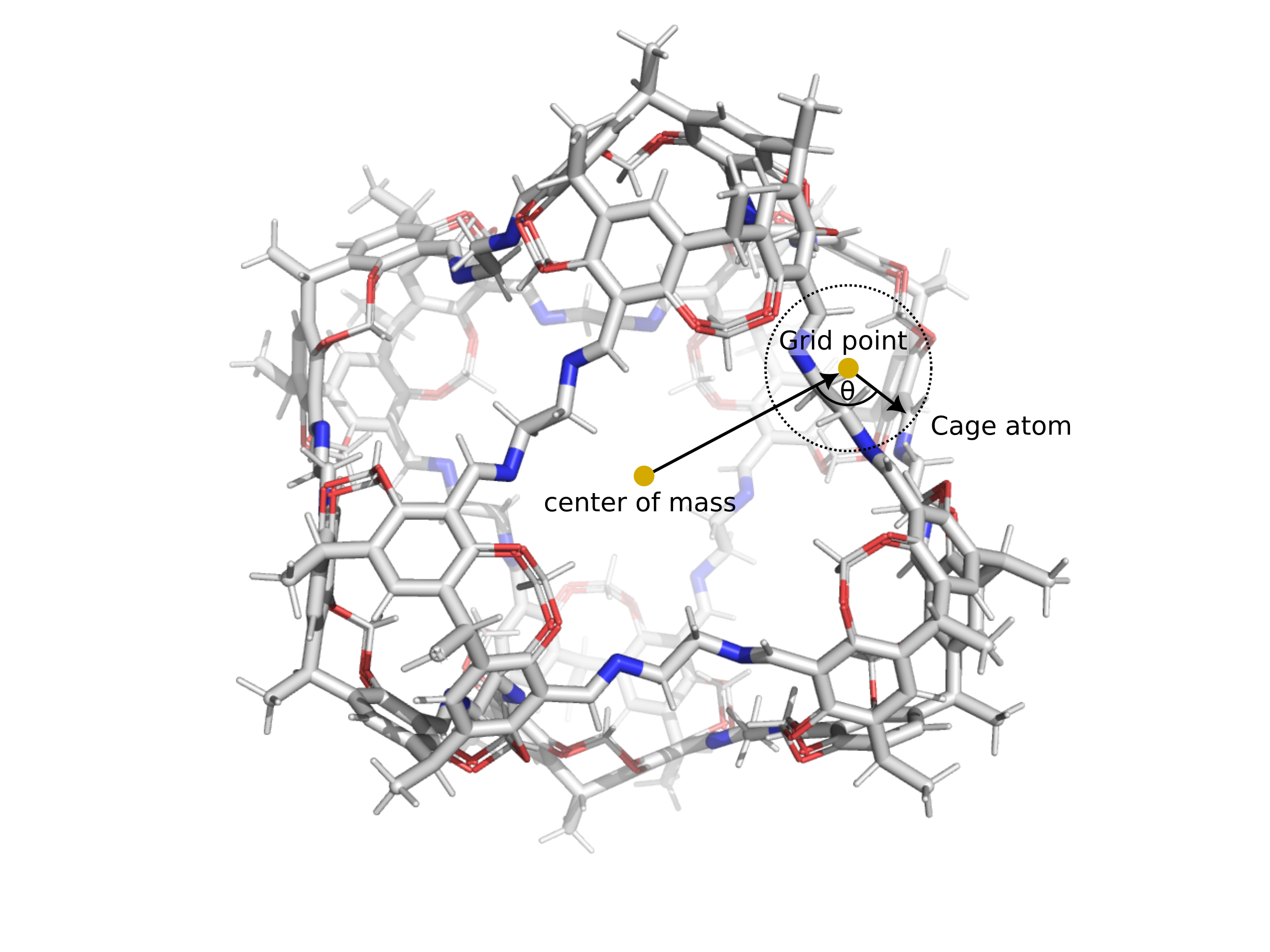

Supplement: Supplementary file 2 — ci4c00355_si_002.zip [file ci4c00355_si_002.zip › CageCavityCalc-v.1.0.5/CageCavityCalc/pic/principle.png]
